# Synthesis, Biological Evaluation, and Structure–Activity Relationships of 4-Aminopiperidines as Novel Antifungal Agents Targeting Ergosterol Biosynthesis

**DOI:** 10.3390/molecules26237208

**Published:** 2021-11-28

**Authors:** Jürgen Krauß, Christoph Müller, Monika Klimt, Leandro Jorquera Valero, José Francisco Martínez, Martin Müller, Karin Bartel, Ulrike Binder, Franz Bracher

**Affiliations:** 1 Department of Pharmacy—Center for Drug Research, Ludwig-Maximilians University Munich, 81377 Munich, Germany; hjkra@cup.uni-muenchen.de (J.K.); christoph.mueller@cup.uni-muenchen.de (C.M.); monika.klimt@cup.uni-muenchen.de (M.K.); 2 Institute of Hygiene and Medical Microbiology, Department of Hygiene, Microbiology and Public Health, Medical University Innsbruck, Schöpfstr. 41, 6020 Innsbruck, Austria; leandro.jorquera01@estudiant.upf.edu (L.J.V.); josefrancisco.martinezh@um.es (J.F.M.); ulrike.binder@i-med.ac.at (U.B.); 3 Department of Pharmacy, Pharmaceutical Biology, Ludwig-Maximilians University Munich, 81377 Munich, Germany; martin.mueller.lmu@gmail.com (M.M.); karin.bartel@cup.uni-muenchen.de (K.B.)

**Keywords:** 4-aminopiperidine, antifungals, reductive amination, *Aspergillus* spp., *Candida* spp., *Mucorales* spp.

## Abstract

The aliphatic heterocycles piperidine and morpholine are core structures of well-known antifungals such as fenpropidin and fenpropimorph, commonly used as agrofungicides, and the related morpholine amorolfine is approved for the treatment of dermal mycoses in humans. Inspired by these lead structures, we describe here the synthesis and biological evaluation of 4-aminopiperidines as a novel chemotype of antifungals with remarkable antifungal activity. A library of more than 30 4-aminopiperidines was synthesized, starting from *N*-substituted 4-piperidone derivatives by reductive amination with appropriate amines using sodium triacetoxyborohydride. Antifungal activity was determined on the model strain *Yarrowia lipolytica*, and some compounds showed interesting growth-inhibiting activity. These compounds were tested on 20 clinically relevant fungal isolates (*Aspergillus* spp., *Candida* spp., *Mucormycetes*) by standardized microbroth dilution assays. Two of the six compounds, 1-benzyl-*N*-dodecylpiperidin-4-amine and *N*-dodecyl-1-phenethylpiperidin-4-amine, were identified as promising candidates for further development based on their in vitro antifungal activity against *Candida* spp. and *Aspergillus* spp. Antifungal activity was determined for 18 *Aspergillus* spp. and 19 *Candida* spp., and their impact on ergosterol and cholesterol biosynthesis was determined. Toxicity was determined on HL-60, HUVEC, and MCF10A cells, and in the alternative in vivo model *Galleria mellonella*. Analysis of sterol patterns after incubation gave valuable insights into the putative molecular mechanism of action, indicating inhibition of the enzymes sterol C14-reductase and sterol C8-isomerase in fungal ergosterol biosynthesis.

## 1. Introduction

**Figure 1:**
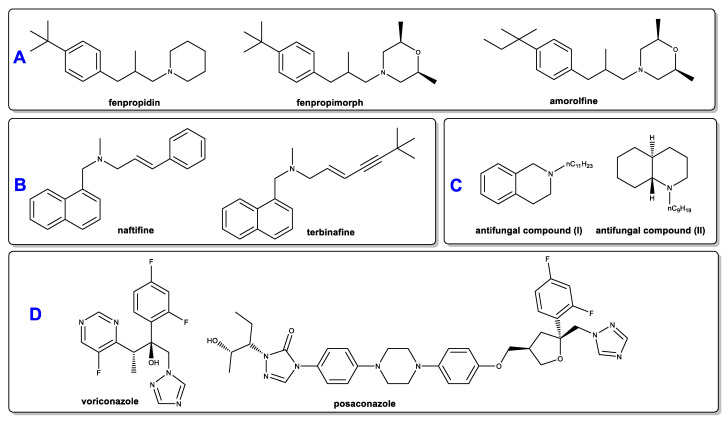
Structures of established antifungals. (**A**) piperidines/morpholines: fenpropidin, fenpropimorph, amorolfine; (**B**) approved allylamines: naftifine, terbinafine; (**C**) antifungal compounds (I) [9] and (II) [10] from our previous work; (**D**) approved azoles: voriconazole, posaconazole.

Fenpropidin and fenpropimorph [1,2] (Figure 1) are well-established antifungals with a broad application in agrochemistry since the 1980s. The structurally related morpholine derivative amorolfine (Loceryl^®^) is used in human dermatology, especially for the treatment of onychomycosis and various local dermal mycoses. The mechanism of action of these antifungals is inhibition of ergosterol biosynthesis. These and related morpholines and piperidines inhibit, to various extents, the enzymes sterol C14-reductase and sterol C8-isomerase of the post-squalene part of ergosterol biosynthesis [3,4]. At physiological pH protonated, the core element for this enzyme inhibition is the morpholine/piperidine ring, which can imitate the carbocationic high-energy intermediates (HEIs) of the conversions catalysed by these two enzymes [5,6,7,8].

In the last decades, an increase in fungal resistance against commonly used antifungals was seen in clinical fungal isolates such as *Aspergillus fumigatus* and *Candida* species; furthermore, an increase in emerging fungal pathogens, such as Mucorales, was observed [11,12,13,14]. Consequently, the antifungal therapy of immunocompromised patients is becoming even more difficult and the need for development of new antifungals is an urgent demand. In parallel to the clinical developments, the demand for novel antifungal agrochemicals is similarly high, as resistance is emerging in this field, too, and a number of old antifungals used in crop protection are meanwhile hampered by safety concerns [15].

One promising strategy in the development of new antifungal compounds with advantages such as higher potency, a broader spectrum, fewer side effects, and a better ecological balance sheet and to break resistance is to start from well-established antifungals as lead structures by means of design and further optimization of hybrids of different active chemotypes [16].

In continuation of our research on the development of antifungals starting from simple aliphatic amines such as benzylamines, (partly) hydrogenated quinolines, and isoquinolines [9,10,17,18] (Figure 1C), we merged essential fragments from these chemotypes and evaluated the 4-aminopiperidine motif as a core structure for novel antifungals. The introduction of a second protonable nitrogen into a sterol biosynthesis inhibitor designed to imitate a carbocationic HEI had shown great benefit in our recent investigations on oxidosqualene cyclase inhibitors [19]. The nature of the residues at both nitrogen atoms of the 4-aminopiperidine core structure was inspired by arylalkylamines (e.g., fenpropidin, Figure 1A), as well as allylamine-type drugs (squalene epoxidase inhibitors) such as naftifine and terbinafine (Figure 1B) on the one side, and medium to long, linear or branched alkyl chains (Figure 1C; see evidence described in ref. [9,10,17]) on the other side.

We expected that fine-tuning of the antifungal activity of this new class of compounds should be possible by systematic modification of both *N*-substituents.

## 2. Results and Discussion

### 2.1. Chemistry

**Scheme 1:**
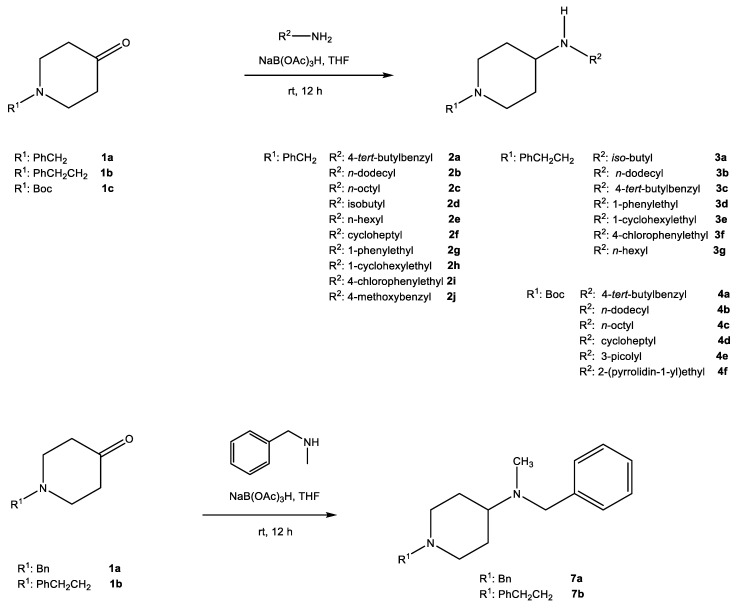
Synthesis of compounds **2a**–**j**, **3a**–**g**, **4a**–**f** and **7a**,**b**.

Commercially available *N*-substituted 4-piperidone derivatives **1a**–**c** were subjected to reductive amination with diverse aliphatic amines, using sodium triacetoxyborohydride as the reducing agent [17,20], to give the secondary amines **2a**–**j**, **3a**–**g**, **4a**–**f** and the tertiary amines **7a**,**b** in moderate to virtually quantitative yields (Scheme 1). In order to ensure stability and sufficient water solubility, all resulting amines could be converted into their corresponding dihydrochlorides (monohydrochlorides in case of the *N*-Boc compounds **4a**–**f**) by precipitation with hydrogen chloride in diethyl ether.

**Scheme 2:**
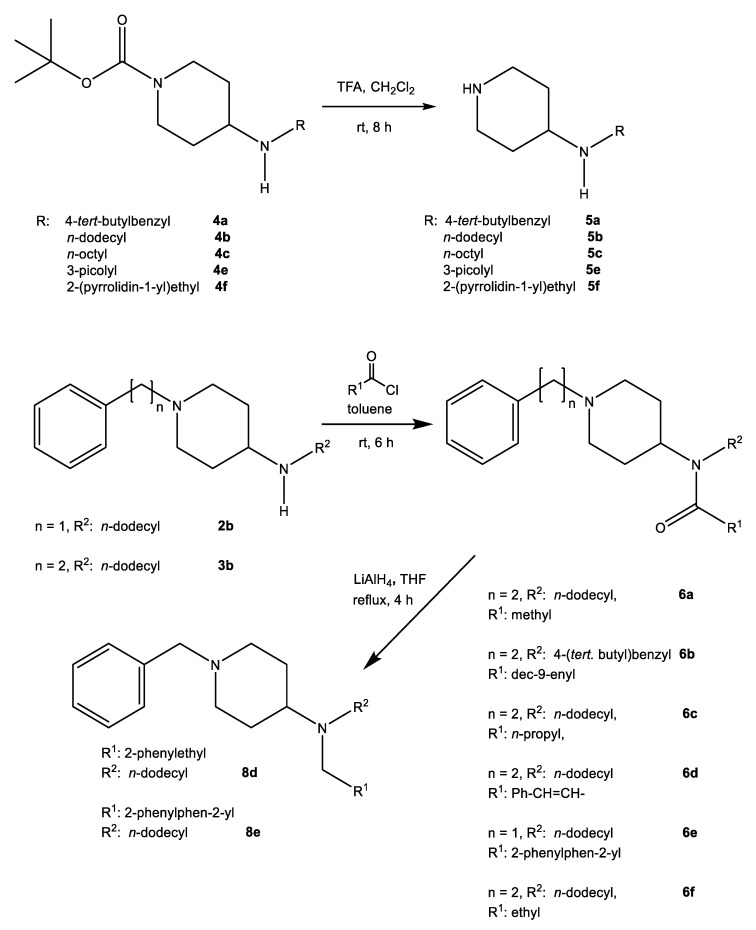
Synthesis of compounds **5a**–**f**, **6a**–**f**, and **8d**,**e**.

The *N*-Boc protecting group of **4a**–**c**, **e**, **f** could be removed by trifluoroacetic acid treatment [21] to give the corresponding unprotected piperidines **5a**–**c**, **e**, **f** in good yields (Scheme 2). Attempted deprotection with hydrogen chloride in diethyl ether [22] resulted only in incomplete conversions in same cases.

In order to investigate the importance of having two protonable nitrogen atoms in the molecules, the secondary amines **2b** and **3b** were reacted with diverse carboxylic acid chlorides (acetyl chloride, 10-undecenoyl chloride, butanoyl chloride, propanoyl chloride, cinnamoyl chloride, and 2-phenylbenzoyl chloride) to give the amides **6a**–**f**. Notably, the amides **6d** and **6e** were reduced with LiAlH_4_ in THF to give the corresponding bulky tertiary amines **8d** and **8e**. The alkene group of **6d** was hydrogenated in the same reaction.

### 2.2. Biology

#### 2.2.1. Screening for In Vitro Antifungal Activity

First, the antifungal activity (minimum inhibitory concentration, MIC) of the resulting compounds was evaluated in an in-house microdilution assay against the non-pathogenic yeast strain *Yarrowia lipolytica* (Supporting Information, Table S1). Furthermore, the compounds causing the highest growth inhibition in *Y. lipolytica* (and **6a**, the acetamide derivative of **3b**) were subjected to an extended antifungal activity screening according to the standardized method of the European Committee of Antifungal Susceptibility Testing [23]. Commonly used antifungal agents (amorolfine, voriconazole, Figure 1) were used for comparison (Table 1).

The compounds **2b**, **3b**, **4b**, and **5b** showed a complete growth inhibition (MIC_100_) in the same range as the reference antifungals amorolfine hydrochloride and voriconazole against the model strain *Yarrowia lipolytica*. For clinically relevant species, complete growth inhibition (80% or 90%) was observed in a species- and strain-dependent manner. The lowest minimal inhibitory concentration (MIC) values were determined for compound **3b** against *Candida* spp. and *Aspergillus* spp. (MIC range 1–4 µg/mL for yeasts and 1–8 µg/mL for Aspergilli). In both groups, MIC values were significantly lower (min. 2 dilution steps) than for amorolfine hydrochloride. Similar reduction in MICs was observed for **2b**. Except for *A. terreus*, confrontation of spores with amide **6a** did not result in a complete growth inhibition for Aspergilli or *Candida* spp. at the concentrations tested. Similarly, no complete growth inhibition was detected for *A. flavus* and *A. fumigatus* for compound **2c**. All other candidates efficiently inhibited growth in these groups. For the group of *Mucormycetes*, MICs were considerable higher than for the yeasts, in many strains not reaching 90% growth inhibition at the concentrations tested. Again, the lowest MICs were obtained for **3b** against this group of fungi (MIC range 4–>16 µg/mL). As *Mucormycetes* exhibit high resistance to commonly used antifungals, having a compound at hand causing complete growth inhibition when applied on spores is a promising result [24].

A number of structure–activity relationships can be deduced from these screening results: Both a benzyl and a phenylethyl substituent at the piperidine nitrogen can lead to high antifungal activity (see compounds **2b** and **3b**), as long as they are combined with *N*-alkyl substituents with more than seven carbon atoms at the 4-amino group. Shorter, branched, or cyclic alkyl residues at the 4-amino group are detrimental to activity; the same holds for most of the arylalkyl residues (except 4-*tert*-butylbenzyl in **2a**). Outstanding antifungal activity was found for the *N*-dodecyl (C_12_) residue (see **2b** and **3b**). In case this residue was attached to the 4-amino group, even compounds that are unsubstituted at the piperidine nitrogen (**5b**) or substituted with a Boc group there (**4b**) showed noteworthy activity. These findings for the *N*-dodecyl residue are in good accordance with SAR detected for antifungal *N*-alkyl perhydroisoquinolines [9] and *N*-alkyl perhydroquinolines [10], ergosterol biosynthesis inhibitors with amorolfine-like mode of action (inhibition of the enzymes sterol C14-reductase and/or sterol C8-isomerase).

Acylation of the secondary exocyclic amino group (**6a**–**f**, Supporting Information, Table S1) led to a virtually complete loss of antifungal activity; the same holds for the introduction of small (**7a,b,** Supporting Information, Table S1) or large residues there (**8d/e,** Supporting Information, Table S1). 

#### 2.2.2. Evaluation of the Antifungal Activity of **2b** and **3b** on Clinical Isolates

The compounds **2b** and **3b** showed the most promising growth-inhibiting activity on *Aspergillus* spp. and *Candida* spp. In the next step, we determined the antifungal activity on a greater collection of clinical isolates of *Aspergillus* spp. (*n* = 18) and *Candida* spp. (*n* = 19), to rule out strain specific differences in antifungal susceptibility (Table 2). In addition to MIC values, the minimal fungicidal concentrations (MFCs) were determined for selected *Candida* and *Aspergillus* isolates. MFCs are used to characterize the antifungal activity either into fungistatic or fungicidal. As seen before in the small set of strains, the MIC values for **2b** and **3b** were lower compared to those for amorolfine hydrochloride. In the case of *C. krusei*, the MIC values for amorolfine hydrochloride were highly variable between the individual isolates while they were consistent for the new substances. Compound **3b** showed lower MICs than **2b**. For the *Aspergillus* spp., MICs could be defined for all strains ranging from 4 µg/mL to 16 µg/mL, while no MIC_90_ was detected for amorolfine hydrochloride at the concentrations tested. Interestingly, while no MFC could be observed for any of the tested species for amorolfine hydrochloride, the new compounds led to a clearly defined MFC (Supporting Information, Figure S1), resulting in no growth/no colony-forming units (CFUs) at concentrations that resembled the MIC or were one dilution step higher than the MIC. These data indicate that contrary to amorolfine hydrochloride, the novel substances do exhibit fungicidal activity on selected *Candida* and *Aspergillus* isolates at concentrations that resemble the MIC, or only one dilution step higher. These data point to a higher antifungal activity of the novel compounds compared to the approved ergosterol biosynthesis inhibitor amorolfine hydrochloride. Rex et al. [25] have already pointed out in a review that MFCs might be the values more relevant to predict clinical outcome compared to solely MIC values.

#### 2.2.3. Evaluation of Toxicity in Human Cell Lines and an Alternative In Vivo Model

In order to evaluate potential development of the compounds as antifungals applied in mammalian systems, toxicity tests were carried out with **2b** and **3b** on 3 different human cell lines in a standard proliferation assay [26]. For this purpose, we used HL-60 cells, HUVEC cells, and MCF10A. HUVEC cells are primary endothelial cells that are commonly used in research to assess cytotoxicity for the blood vessel system [27], MCF10A cells represent a healthy epithelial cell line that is frequently used in cytotoxicity studies [28], and HL-60 cells are used as an alternative to primary neutrophils and used to assess cytotoxicity for immune cells [29]. By combining cytotoxicity studies in these cell lines, we gained data on general toxicity of the compounds in human cells. The antifungals amorolfine hydrochloride (used as topical formulation), posaconazole, and voriconazole (both used for the treatment of invasive fungal infections) were used as reference drugs. The results are shown in Table 3.

Compared to the reference drugs amorolfine hydrochloride and voriconazole, compounds **2b** and **3b** showed enhanced cytotoxicity. In HL-60 cells, the IC_50_ value for posaconazole was in the same range as compound **2b** and **3b**.

**Figure 2:**
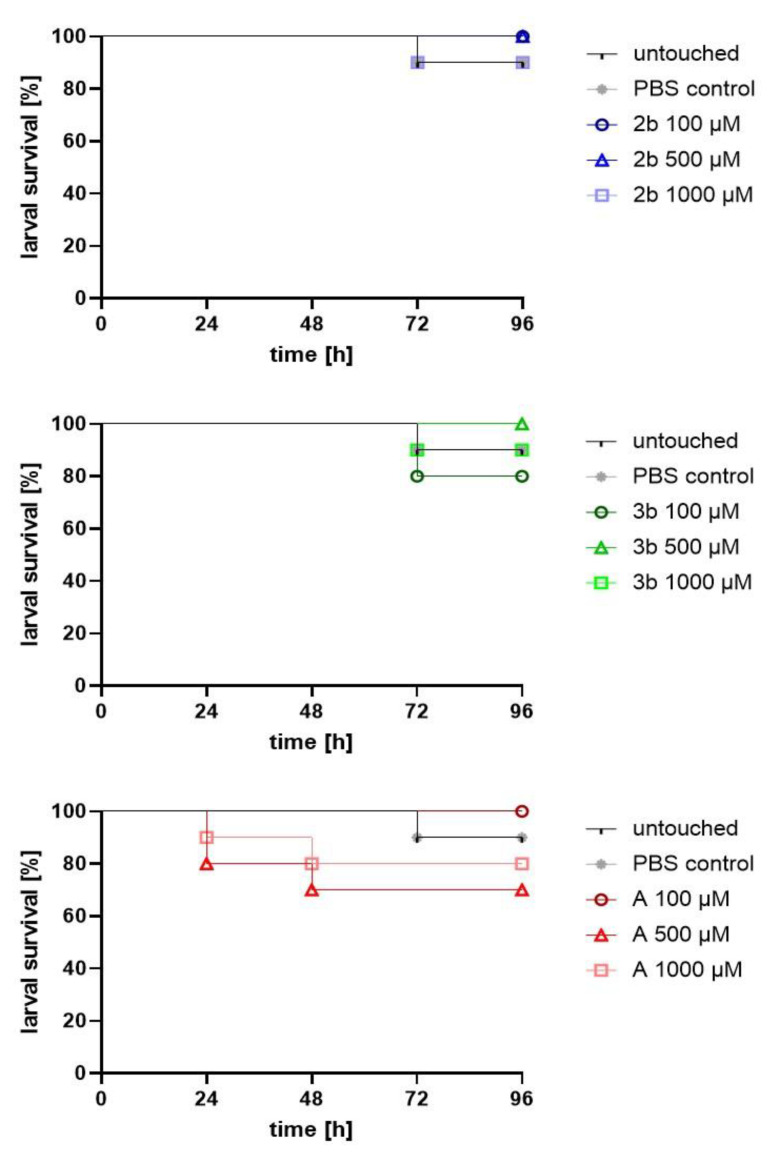
Impact of antifungal compounds **2b**, **3b** and amorolfine hydrochloride (**A**) on the survival of *Galleria mellonella* larvae. The Kaplan Meyer curves represent average survival rates of 40 larvae per sample (2 independent experiments). Each larva was injected with 20 µL of the respective antifungal solution, and dilutions were made in PBS. Untouched larvae and larvae injected with PBS served as controls. Test compound concentration was chosen according to MIC data: 100 µM represents the MIC/g larvae for **2b** and **3b**; 500 µM, the MIC for amorolfine hydrochloride; and 1000 µM 10-fold, the MIC of **2b** and **3b**.

*Galleria mellonella* larvae have been widely used as an alternative infection in vivo model for fungal diseases [30] and have also been utilized to study the in vivo activity and toxicity of commonly used or novel antimicrobial agents [31]. Therefore, the larval system was used to determine a potential impact on survival of larvae injected with three different doses of either compound **2b** or **3b**; amorolfine hydrochloride was used for comparison. All dilutions were made in PBS, which served as negative control. The Kaplan Meyer curves (Figure 2) show that none of the compounds tested significantly reduced survival compared to PBS control (log rank test; *p* < 0.05), indicating no toxic activity in this model system. These results are promising for the further testing of the in vivo activity of the new compounds in animal models. Nevertheless, the survival data presented do not give insight into the potential impact these compounds might have on the larval immune system, the stability of the compounds in the larval hemolymph, and their tissue availability [32]. Further assays are necessary to investigate in detail (a) whether the larval model is suitable to test the in vivo efficiency of these novel compounds, and (b) whether treatment leads to an increase in the survival of fungus-infected larvae.

#### 2.2.4. Identification of Target Enzymes in Ergosterol and Cholesterol Biosynthesis

The orienting tests for antifungal activity shown in Section 2.2.1 clearly indicated SAR related to those identified by us for antifungal *N*-alkyl perhydroisoquinolines [9] and *N*-alkyl perhydroquinolines [10] before. In the isoquinoline series, we found that even almost equipotent compounds can inhibit different target enzymes. In contrast to amorolfine and related *N*-alkylmorpholines/-piperidines, which inhibit both sterol C8-iso-merase and sterol C14-reductase, these compounds were found to be inhibitors of either one or the other of these two enzymes. The *N*-alkyl perhydroquinolines, however, exclusively inhibit the enzyme sterol C8-isomerase. This prompted us to investigate the effect of the top compounds **2b** and **3b** from this investigation on the post-lanosterol part of ergosterol biosynthesis using a cellular assay [3]. 

In total, 15 different sterols were identified in *A. fumigatus* (mold), *C. albicans* (yeast), and *C. glabrata* (yeast) confronted with sublethal concentrations of amorolfine hydrochloride (**A**), **2b** and **3b** (Table 4). To calibrate target identification, we used the well-established antifungal amorolfine hydrochloride. Under amorolfine treatment, a more than 10-fold accumulation of ergosta-8,14,24(28)-trien-3β-ol, ergosta-5,8,24(28)-trien-3β-ol, and ergosta-8,24(28)-dien-3β-ol was detected compared to the control sample in all strains. An accumulation of ergosta-8,14,24(28)-trien-3β-ol indicates an inhibition of the enzyme sterol C14-reductase, and an accumulation of ergosta-5,8,24(28)-trien-3β-ol and ergosta-8,24(28)-dien-3β-ol indicates an inhibition of sterol C8-isomerase [3,4]. As expected, the mode of action of amorolfine is a dual inhibition of sterol C14-reductase and sterol C8-isomerase. Although there was no accumulation of lichesterol (ergosta-5,8,22-trien-3β-ol), the marker sterol for sterol C8-isomerase [3], this is due to the additional upstream enzyme inhibition of sterol C14-reductase, which leads to an accumulation of sterols with a remaining double bond at C14/15. The appearance of hydroxyfecosterol in both yeast strains can be explained by inhibition or improper working of sterol C8-isomerase similar to the appearance of 14-methylergosta-8,24(28)-dien-3β,6α-diol under azole treatment [3,4,33,34]. 

Compound **2b** showed a similar sterol pattern in *A. fumigatus* and *C. glabrata* as amorolfine, indicating a similar mode of action. In *C. albicans*, the low MIC values (Table 2) cannot fully be explained by a moderate accumulation (less than ten-fold) of ergosta-5,7,24(28)-trien-3β-ol, which is a marker sterol for inhibition of sterol C5-desaturase [3]. There was no hint for inhibition of the enzyme sterol C14-reductase or the enzyme sterol C8-isomerase in this yeast. Compound **3b** showed similar results as **2b** in *C. albicans*. A dual inhibition of sterol C14-reductase and sterol C8-isomerase was only detected in *C. glabrata.* In *A. fumigatus*, an accumulation of ergosta-8,24(28)-dien-3β-ol was detected, which indicates inhibition of sterol C8-isomerase.

Since ergosterol biosynthesis in fungi and cholesterol biosynthesis in humans are very similar and share numerous closely related enzymes, we investigated the effects of these compounds on cholesterol biosynthesis in a cellular assay [35] as well. 

In the cholesterol biosynthesis assay, we investigated, in addition to the most active compounds **2b**/**3b** (Table 5), a number of additional compounds (**2c**, **2f**, **2g**, **3c, 4b**, **4c**, **5b**, and **6b**; see Supporting Information, Table S2) from this library in order to get a first insight into SAR on this alternative target as well. Not unexpectedly, most of the compounds showed impact on enzymes of the post-lanosterol part of this biosynthesis, with multi-enzyme inhibition dominating at higher concentrations and inhibition of both sterol C8-isomerase and sterol C14-reductase at lower concentrations. The analysis of the sterol pattern of the reference inhibitor amorolfine hydrochloride (Table S2) showed only an accumulation of cholesta-8,14-dien-3β-ol, which indicates inhibition of sterol C14-reductase and sterol C8-isomerase at every test level (0.1 µM, 1 µM, 10 µM). Antifungal compound **2b** (Table 5) as well as **2f** (Supporting Information, Table S2) showed inhibition of sterol C8-isomerase and sterol C14-reductase at 0.1 µM, and a multi-enzyme inhibition at higher concentrations. The other strong antifungal compound, **3b** (Table 5), was identified as a multi-enzyme inhibitor at 1 µM (inactive at 0.1 µM, toxic at 10 µM). Only compound **4c** (Supporting Information, Table S2) was selective for sterol C14-reductase and sterol C8-isomerase at 1 µM and 10 µM (inactive at 0.1 µM). Notably, compound **2g** did not show any effect on cholesterol biosynthesis. 

## 3. Conclusions

A library of more than 30 novel 4-aminopiperidines was prepared by reductive amination of 4-piperidone derivatives with a broad variety of aliphatic amines. A screening on the model yeast *Yarrowia lipolytica* disclosed that compounds **2b** and **3b** are almost equipotent to established antifungals. In tests on clinically relevant species (*Candida* spp., *Aspergillus* spp., *Mucormycetes*), compounds **2b** and **3b** favourably compared with the approved antifungals amorolfine and voriconazole. Analysis of SAR revealed that the combination of a benzyl or phenylethyl residue at the piperidine nitrogen with an *n*-dodecyl residue at the 4-amino group is most beneficial to enhance antifungal activity. The two top compounds **2b** and **3b** are, as expected, inhibitors of the fungal ergosterol biosynthesis enzymes sterol C14-reductase and sterol C8-isomerase. However, additional molecular targets cannot be excluded. Determination of MIC values and minimal fungicidal concentrations (MFCs) for selected *Candida* and *Aspergillus* isolates revealed that, contrary to amorolfine, the novel substances do exhibit fungicidal activity. Antifungal activity was further determined on a greater collection of clinical isolates of *Aspergillus* spp. and *Candida* spp., and here **3b** was clearly superior to amorolfine. The top compounds **2b** and **3b** exhibit cytotoxicity on human cell lines, but not on the *Galleria mellonella* larvae in an alternative (more complex) test system. The moderate cytotoxicity on human cell lines can in part be explained by inhibition of cholesterol biosynthesis. This aspect needs deeper investigation in the future if these or related compounds are considered for further development. 

The 4-aminopiperidine core has been identified as an interesting lead structure for development of novel antifungals. It might be the basis for the development of antifungals targeting (at least among others) ergosterol biosynthesis. Due to the easy availability starting from cheap building blocks, application as agrofungicides might be considered as well.

## 4. Experimental

### 4.1. Chemistry

#### 4.1.1. General

All solvents used were of HPLC grade or *p.a*. grade and/or purified according to standard procedures. Chemical reagents were purchased from Sigma Aldrich (Schnelldorf, Germany) and Acros (Geel, Belgium). IR spectra: Jasco (Pfungstadt, Germany) FT/IR 4600 series (KBr pellet method or ATR Zn/Se); MS: Hewlett Packard MS-Engine (Agilent, Santa Clara, CA, USA), electron ionization (EI) 70 eV, chemical ionization (CI) with CH_4_ (300 eV); MS spectra: Thermo Q Exactive GC Orbitrap or Finnigan MAT 95 spectrometer; HR-ESI-MS spectra: Thermo Finnigan LTQ FT; NMR: Avance III HD 400 MHz Bruker BioSpin (^1^H: 400 MHz, ^13^C: 100 MHz); 500 MHz Avance III HD 500 MHz Bruker BioSpin (^1^H: 500 MHz, ^13^C: 125 MHz); melting points: Büchi Melting Point B-540 (not corrected; Büchi Labortechnik GmbH, Essen, Germany); flash column chromatography (FCC): silica gel 60 (230–400 mesh, E. Merck, Darmstadt); polarimeter: Perkin Elmer 241 (Perkin Elmer, Rodgau, Germany).

##### General Procedure 1 (Reductive Amination)

In total, 1.0 mmol of the ketone and 1.5 mmol of the amine were dissolved in 20 mL dry THF, and 2.0 mmol sodium triacetoxyborohydride was added. The suspension was stirred at room temperature for 12 h. Then, 20 mL of a saturated aqueous NaHCO_3_ solution was added and the mixture was extracted with ethyl acetate (3 × 20 mL). The combined organic layers were dried over Na_2_SO_4_ and the solvent was evaporated. The residue was purified by flash column chromatography (ethyl acetate:triethylamine 10:1).

##### General Procedure 2 (Cleavage of Boc-Protecting Group)

The Boc-protected amine (1.0 mmol) was dissolved in 20 mL dichloromethane, and 10 mL trifluoroacetic acid was added. The solution was stirred at room temperature for 8 h and then 20 mL 10% aqueous sodium hydroxide solution was added. The mixture was extracted with dichloromethane (3 × 20 mL) and the combined organic layers were dried over Na_2_SO_4_. The solvent was evaporated and the residue was purified by flash column chromatography (ethyl acetate:triethylamine 10:1) [10].

##### General Procedure 3 (Synthesis of Amides)

In total, 1.0 mmol of the amine was dissolved in 20 mL toluene and 1.2 mmol of the acid chloride was added. After addition of 3.0 mL triethylamine, the mixture was stirred at room temperature for 6 h. The solvent was evaporated and the residue was dissolved in 20 mL 2 M aqueous sodium hydroxide solution and extracted with ethyl acetate (3 × 20 mL). The combined organic layers were dried over Na_2_SO_4_ and the solvent was evaporated. The residue was purified by flash column chromatography (ethyl acetate:triethylamine 10:1).

*1-Benzyl-N-(4-(tert-butyl)benzyl)piperidin-4-amine* (**2a**): The compound was prepared according to general procedure 1 from 568 mg (3.0 mmol) 1-benzyl-4-piperidone, 734 mg (4.5 mmol) 4-*tert*-butylbenzylamine, and 1.34 g (6.0 mmol) sodium triacetoxyborohydride to give 707 mg (70%) of **2a** as a colourless oil. ^1^H-NMR (400 MHz, dichloromethane-*d*_2_) δ 7.35–7.28 (m, 6 H, 6 arom. CH), 7.26–7.20 (m, 3 H, 3 arom. CH), 3.74 (s, 2 H; CH_2_), 3.46 (s, 2 H, CH_2_), 2.85–2.73 (m, 2 H, 2 CH_2_), 2.56–2.44 (m, 1 H, CH), 2.07–1.96 (m, 2 H, 2 CH_2_), 1.89–1.80 (m, 2 H, 2 CH_2_), 1.48–1.24 (m, 2 H, 2 CH_2_), 1.30 (s, 9 H, 3 CH_3_). ^13^C-NMR (100 MHz, dichloromethane-*d*_2_) δ 150.91 (quat. C), 140.47 (quat. C), 139.55 (quat. C), 130.27 (2 arom. CH), 129.36 (2 arom. CH), 128.97 (2 arom. CH), 128.05 (arom. CH), 126.44 (2 arom. CH), 64.31 (CH_2_), 55.68 (CH), 53.70 (CH_2_), 51.68 (2 CH_2_), 35.61 (quat. C), 34.18 (2 CH_2_), 32.44 (3 CH_3_). IR (KBr) ν (cm^−1^) = 2956, 2867, 2800, 1455, 1363, 1268, 1111, 819, 791, 737, 698. MS (EI): *m/z*: 336 (M^+^, 2), 245 (5), 189 (37), 173 (65), 91 (100). HRMS (EI) calcd. for C_23_H_32_N_2_: 336.2567. Found: 336.2561.

*1-Benzyl-N-dodecylpiperidin-4-amine* (**2b**): The compound was prepared according general procedure 1 from 568 mg (3.0 mmol) 1-benzyl-4-piperidone, 834 mg (4.5 mmol) *n*-dodecylamine and 1.34 g (6.0 mmol) sodium triacetoxyborohydride to give 699 mg (65%) of **2b** as a colourless oil. ^1^H-NMR (400 MHz, dichloromethane-*d*_2_) δ 7.34–7.14 (m, 5 H, 5 arom. CH), 3.45 (s, 2 H, CH_2_), 2.84–2.75 (m, 2 H, 2 CH_2_), 2.57 (t, *J* = 7.0 Hz, 2 H, CH_2_), 2.42 (tt, *J* = 10.5, 4.1 Hz, 1 H, CH), 1.99 (td, *J* = 11.7, 2.3 Hz, 2 H, CH_2_), 1.85–1.74 (m, 2 H, 2 CH_2_), 1.48–1.37 (m, 2 H, CH_2_), 1.34–1.20 (m, 20 H, 10 CH_2_), 0.87 (t, *J* = 6.9 Hz, 3 H, CH_3_). ^13^C-NMR (100 MHz, dichloromethane-*d*_2_) δ 140.41 (quat. C), 130.28 (2 arom. CH), 129.36 (2 arom. CH), 128.07 (arom. CH), 64.28 (CH_2_), 56.35 (CH), 53.77 (2 CH_2_), 48.00 (CH_2_), 33.95 (2 CH_2_), 33.24 (CH_2_), 31.55 (CH_2_), 30.99 (CH_2_), 30.96 (3 CH_2_), 30.90 (CH_2_), 30.67 (CH_2_), 28.73 (CH_2_), 24.01 (CH_2_), 15.20 (CH_3_). IR (KBr) ν (cm^−1^) = 2915, 2849, 2797, 1469, 1453, 1365, 1345, 1125, 1112, 792, 736, 727, 716, 694. MS (EI) *m/z*: 358 (M^+^, 2), 267 (22), 173 (100), 91 (70). HRMS (EI) calcd. for C_24_H_42_N_2_: 358.3348. Found: 358.3345.

*1-Benzyl-N-octylpiperidin-4-amine* (**2c**): The compound was prepared according to general procedure 1 from 568 mg (3.0 mmol) 1-benzyl-4-piperidone, 581 mg (4.5 mmol) *n*-octylamine and 1.34 g (6.0 mmol) sodium triacetoxyborohydride to give 544 mg (60%) of **2c** as a colourless oil. ^1^H-NMR (500 MHz, chloroform-*d*) δ 7.28–7.21 (m, 4 H, 4 arom. CH), 7.18–7.14 (m, 1 H, arom. CH), 3.42 (s, 2 H, CH_2_)_,_ 2.84–2.67 (m, 2 H, 2 CH_2_), 2.52 (t, *J* = 7.3 Hz, 2 H, CH_2_), 2.36 (dt, *J* = 10.6, 6.6 Hz, 1 H, CH), 1.99–1.85 (m, 2 H, 2 CH_2_), 1.83–1.69 (m, 2 H, 2 CH_2_), 1.44–1.35 (m, 2 H, 2 CH_2_), 1.35–1.26 (m, 2 H, CH_2_), 1.26–1.14 (m, 10 H, 5 CH_2_), 0.81 (t, *J* = 6.9 Hz, 3 H, CH_3_). MS (EI) m/z = 302 (M^+^, 3), 211 (20), 173 (100), 146 (16), 91 (88). ^13^C-NMR (100 MHz, chloroform-*d*) δ 138.67 (quat. C), 129.08 (2 arom. CH), 128.13 (2 arom. CH), 126.87 (arom. CH), 63.10 (CH_2_), 55.07 (CH), 52.57 (2 CH_2_), 46.92 (CH_2_), 32.90 (2 CH_2_), 31.84 (CH_2_), 30.51 (CH_2_), 29.54 (CH_2_), 29.28 (CH_2_), 27.46 (CH_2_), 22.67 (CH_2_), 14.11 (CH_3_). IR (KBr) ν (cm^−1^) = 2924, 2853, 2799, 1466, 1454, 1365, 1119, 792, 735, 697. MS (EI) m/z: 300 (M^+^, 0.2), 299 (1), 211 (13), 173 (100), 158 (26), 146 (14), 91 (72), 82 (20). HRMS (EI) calcd. for C_20_H_32_N_2_ (M^+^-2): 300.2566. Found: 300.2514.

*1-Benzyl-N-isobutylpiperidin-4-amine* (**2d**): The compound was prepared according to general procedure 1 from 568 mg (3.0 mmol) 1-benzyl-4-piperidone, 329 mg (4.5 mmol) isobutylamine and 1.34 g (6.0 mmol) sodium triacetoxyborohydride to give 502 mg (68%) of **2d** as a colourless oil. ^1^H-NMR (400 MHz, chloroform-*d*) δ 7.29–7.06 (m, 5 H, 5 arom. CH), 3.42 (s, 2 H, CH_2_), 2.85–2.67 (m, 2 H, CH_2_),2.39–2.29 (m, 1 H, CH), 2.35 (d, *J* = 6.8 Hz, 2 H, CH_2_), 1.99–1.89 (m, 2 H, CH_2_), 1.82–1.72 (m, 2 H, CH_2_), 1.63 (hept, *J* = 6.7 Hz, 1 H, CH), 1.31 (qd, *J* = 11.4, 3.5 Hz, 2 H, CH_2_), 0.83 (d, *J* = 6.6 Hz, 6 H, 2 CH_3_). ^13^C-NMR (100 MHz, chloroform-d) δ 138.65 (quat. C), 129.10 (2 arom. CH), 128.14 (2 arom. CH), 126.89 (arom. CH), 63.10 (CH_2_), 55.03 (CH), 54.90 (2 CH_2_), 52.55 (CH_2_), 32.86 (2 CH_2_), 28.57 (CH), 20.76 (2 CH_3_). IR (KBr) ν (cm^−1^) = 2948, 2935, 2799, 1467, 1454, 1365, 1117, 979, 791. MS (EI) *m/z*: 246 (M^+^, 4), 173 (73), 146 (27), 91 (100), 82 (16). HRMS (EI) calcd. for C_13_H_19_N_2_ (M^+^-C_3_H_7_): 203.1548. Found: 203.1516.

*1-Benzyl-N-hexylpiperidin-4-amine* (**2e**): The compound was prepared according to general procedure 1 from 568 mg (3.0 mmol) 1-benzyl-4-piperidone, 455 mg (4.5 mmol) *n*-hexylamine and 1.34 g (6.0 mmol) sodium triacetoxyborohydride to give 584 mg (71%) of **2e** as a colourless oil. ^1^H-NMR (500 MHz, chloroform-*d*) δ 7.27–7.22 (m, 4 H, 4 arom. CH), 7.19–7.14 (m, 1 H, arom. CH), 3.42 (s, 2 H, CH_2_), 2.82–2.73 (m, 2 H, 2 CH_2_), 2.53 (t, *J* = 7.2 Hz, 2 H, CH_2_), 2.36 (tt, *J* = 10.5, 4.1 Hz, 1 H, CH), 1.94 (td, *J* = 11.7, 2.3 Hz, 2 H, 2 CH_2_), 1.83–1.69 (m, 2 H, 2 CH_2_), 1.43–1.36 (m, 2 H, CH_2_), 1.35–1.28 (m, 2 H, 2 CH_2_), 1.26–1.16 (m, 6 H, 3 CH_2_), 0.81 (t, *J* = 6.9 Hz, 3 H, CH_3_). ^13^C-NMR (100 MHz, chloroform-*d*) δ 138.63 (quat. C), 129.10 (2 arom. CH), 128.14 (2 arom. CH), 126.89 (arom. CH), 63.10 (CH_2_), 55.06 (CH), 52.56 (2 CH_2_), 46.91 (CH_2_), 32.86 (2 CH_2_), 31.79 (CH_2_), 30.46 (CH_2_), 27.14 (CH_2_), 22.63 (CH_2_), 14.06 (CH_3_). IR (KBr) ν (cm^−1^) = 2924, 2853, 2797, 1466, 1454, 1365, 1342, 1117, 1072, 1029, 972, 792. MS (EI) *m/z*: 274 (M^+^, 4), 173 (99), 146 (17), 91 (100). HRMS (EI) calcd. for C_18_H_30_N_2_: 274.2409. Found: 274.2418.

*1-Benzyl-N-cycloheptylpiperidin-4-amine* (**2f**): The compound was prepared according to general procedure 1 from 568 mg (3.0 mmol) 1-benzyl-4-piperi-done, 509 mg (4.5 mmol) cycloheptylamine and 1.34 g (6.0 mmol) sodium triacetoxyborohydride to give 860 mg (100%) of **2f** as a colourless oil. ^1^H-NMR (400 MHz, chloroform-*d*) δ 7.30 (d, *J* = 4.4 Hz, 4 H, 4 arom. CH), 7.26–7.21 (m, 1 H, 1 arom. CH), 3.49 (s, 2 H, CH_2_), 2.90–2.80 (m, 2 H, 2 CH_2_), 2.76 (tt, *J* = 8.1, 4.0 Hz, 1 H, CH), 2.51 (tt, *J* = 10.6, 4.0 Hz, 1 H, CH), 2.01 (td, *J* = 11.7, 2.3 Hz, 2 H, 2 CH_2_), 1.89–1.71 (m, 4 H, 3 CH_2_), 1.70–1.59 (m, 2 H, 2 CH_2_), 1.60–1.45 (m, 4 H, 2 CH_2_), 1.46–1.22 (m, 6 H, 3 CH_2_). ^13^C-NMR (100 MHz, chloroform-*d*) δ 138.63 (quat. C), 129.09 (2 arom. CH), 128.13 (2 arom. CH), 126.88 (arom. CH), 63.10 (CH_2_), 55.16 (CH), 52.77 (2 CH_2_), 51.71 (CH), 35.46 (2 CH_2_), 33.38 (2 CH_2_), 28.13 (2 CH_2_), 24.49 (2 CH_2_). MS (EI) *m/z*: 286 (M^+^, 4), 173 (98), 146 (6), 91 (100). HRMS (EI) calcd. for C_19_H_30_N_2_ (M^+^): 286.2409. Found: 286.2403.

*(S)-1-Benzyl-N-(1-phenylethyl)piperidin-4-amine* (**2g**): The compound was prepared according to general procedure 1 from 568 mg (3.0 mmol) 1-benzyl-4-piperidone, 545 mg (4.5 mmol) (*S*)-α-methylbenzenemethanamine and 1.34 g (6.0 mmol) sodium triacetoxyborohydride to give 730 mg (83%) of **2g** as a colourless oil. ^1^H-NMR (400 MHz, chloroform-*d*) δ 7.35–7.17 (m, 10 H, 10 arom. CH), 3.94 (q, *J* = 6.6 Hz, 1 H, CH), 3.44 (s, 2 H, CH_2_), 2.84–2.70 (m, 2 H, 2 CH_2_), 2.30 (tt, *J* = 10.5, 4.0 Hz, 1 H, CH), 1.99–1.83 (m, 3 H, 3 CH_2_), 1.72–1.62 (m, 1 H, CH_2_), 1.44–1.24 (m, 2 H, 2 CH_2_), 1.31 (d, *J* = 6.6 Hz, 3 H, CH_3_). ^13^C-NMR (100 MHz, chloroform-*d*) δ 146.19 (quat. C), 138.60 (quat. C), 129.10 (2 arom. CH), 128.38 (2 arom. CH), 128.12 (2 arom. CH), 126.87 (arom. CH), 126.75 (arom. CH), 126.50 (2 arom. CH), 63.07 (CH_2_), 54.48 (CH), 52.59 (CH_2_), 52.41 (CH_2_), 51.95 (CH), 33.66 (CH_2_), 32.57 (CH_2_), 25.11 (CH_3_). IR (KBr) ν (cm^−1^) = 2924, 2799, 2759, 1493, 1467, 1451, 1366, 1343, 1117, 793, 761, 737, 698. MS (EI) *m/z*: 295 (M^+^+H, 0.5), 189 (64), 175 (17), 172 (45), 158 (18), 146 (21), 105 (21), 91 (100). HRMS (EI) calcd. for C_20_H_27_N_2_ (M^+^ + H): 295.2174. Found: 295.2170. $\propto \frac{20}{D}$ = −47.7° (1.525, CHCl_3_).

*(S)-1-Benzyl-N-(1-cyclohexylethyl)piperidin-4-amine* (**2h**): The compound was prepared according to general procedure 1 from 568 mg (3.0 mmol) 1-benzyl-4-piperidone, 572 mg (4.5 mmol) (*S*)-(+)-1-cyclohexylethylamine and 1.34 g (6.0 mmol) sodium triacetoxyborohydride to give 700 mg (78%) of **2h** as a colourless oil. ^1^H-NMR (400 MHz, chloroform-*d*) δ 7.33–7.27 (m, 4 H, 4 arom. CH), 7.27–7.17 (m, 1 H, arom. CH), 3.49 (s, 2 H, CH_2_), 2.88–2.77 (m, 2 H, 2 CH_2_), 2.57–2.42 (m, 2 H, 2 CH), 2.01 (qd, *J* = 11.5, 2.6 Hz, 2 H, 2 CH_2_), 1.89–1.59 (m, 7 H, 6 CH_2_, CH), 1.45–1.05 (m, 6 H, 6 CH_2_), 1.06–0.87 (m, 2 H, 2 CH_2_), 0.95 (d, *J* = 6.4 Hz, 3 H, CH_3_). ^13^C-NMR (100 MHz, chloroform-*d*) δ 138.72 (quat. C), 129.09 (2 arom. CH), 128.13 (2 arom. CH), 126.86 (arom. CH), 63.13 (CH_2_), 53.93 (CH), 52.73 (CH_2_), 52.61 (CH_2_), 51.99 (CH), 43.28 (CH), 33.80 (CH_2_), 32.96 (CH_2_), 30.01 (CH_2_), 28.17 (CH_2_), 26.79 (CH_2_), 26.65 (CH_2_), 26.51 (CH_2_), 17.70 (CH_3_). IR (KBr) ν (cm^−1^) = 2921, 2849, 1449, 1366, 1264, 1109, 1072, 890, 792, 735, 697. MS (EI) *m/z*: 301 (M^+^ + H, 0.1), 217 (16), 172 (56), 146 (37), 91 (100). HRMS (EI) calcd. for C_20_H_33_N_2_ (M^+^ + H): 301.2644. Found: 301.2638. $\propto \frac{20}{D}$ = 14.4° (c = 2.33, CHCl_3_).

*1-Benzyl-N-(4-chlorophenethyl)piperidin-4-amine* (**2i**): The compound was prepared according to general procedure 1 from 568 mg (3.0 mmol) 1-benzyl-4-piperidone, 700 mg (4.5 mmol) 4-chlorophenylethylamine and 1.34 g (6.0 mmol) sodium triacetoxyborohydride to give 738 mg (75%) of **2i** as a pale yellow oil. ^1^H-NMR (400 MHz, chloroform-*d*) δ 7.32–7.28 (m, 4 H, 4 arom. CH), 7.27–7.21 (m, 3 H, 3 arom. CH), 7.13 (d, *J* = 8.5 Hz, 2 H, 2 arom. CH), 3.48 (s, 2 H, CH_2_), 2.90–2.79 (m, 4 H, 3 CH_2_), 2.75 (t, *J* = 7.0 Hz, 2 H, CH_2_), 2.50–2.40 (m, 1 H, CH), 2.00 (td, *J* = 11.2, 1.9 Hz, 2 H, 2 CH_2_), 1.86–1.77 (m, 2 H, 2 CH_2_), 1.35 (qd, *J* = 12.2, 11.7, 3.8 Hz, 2 H, 2 CH_2_). ^13^C-NMR (100 MHz, chloroform-*d*) δ 138.62 (quat. C), 138.57 (quat. C), 131.89 (quat. C), 130.01 (2 arom. CH), 129.06 (2 arom. CH), 128.54 (2 arom. CH), 128.15 (2 arom. CH), 126.90 (arom. CH), 63.07 (CH_2_), 54.88 (CH), 52.45 (2 CH_2_), 47.93 (CH_2_), 36.06 (CH_2_), 32.80 (2 CH_2_). IR (KBr) ν (cm^−1^) = 2942, 2883, 2800, 1492, 1363, 1091, 1015, 972, 808, 738, 698. MS (EI) *m/z*: 330 (M^+^, 38), 328 (M^+^, 96), 299 (20), 289 (100), 271 (28). HRMS (EI) calcd. for C_20_H_26_ClN_2_ (M^+^ + H): 329.1785. Found: 329.1779.

*1-Benzyl-N-(4-methoxybenzyl)piperidin-4-amine* (**2j**): The compound was prepared according to general procedure 1 from 379 mg (2.0 mmol) 1-benzyl-4-piperidone, 412 mg (3.0 mmol) 4-methoxybenzylamine and 892 mg (4.0 mmol) sodium triacetoxyborohydride to give 610 mg (98%) of **2j** as a pale yellow oil. ^1^H-NMR (400 MHz, chloroform-*d*) δ 7.33–7.29 (m, 4 H, 4 arom. CH), 7.25–7.20 (m, 1 H, arom. CH), 7.23 (d, *J* = 8.7 Hz, 2 H, 2 arom. CH), 6.85 (d, *J* = 8.7 Hz, 2 H, 2 arom. CH), 3.79 (s, 3 H, OCH_3_), 3.74 (s, 2 H, CH_2_), 3.49 (s, 2 H, CH_2_), 2.84 (dt, *J* = 11.6, 2.8 Hz, 2 H, 2 CH_2_), 2.50 (tt, *J* = 10.5, 4.1 Hz, 1 H, CH), 2.01 (td, *J* = 11.6, 2.3 Hz, 2 H, 2 CH_2_), 1.91–1.82 (m, 2 H, 2 CH_2_), 1.49–1.35 (m, 2 H, CH_2_). ^13^C-NMR (100 MHz, chloroform-*d*) δ 158.54 (quat. C), 138.63 (quat. C), 132.94 (quat. C), 129.20 (2 arom. CH), 129.10 (2 arom. CH), 128.14 (2 arom. CH), 126.89 (arom. CH), 113.80 (2 arom. CH), 63.12 (CH_2_), 55.28 (OCH_3_), 54.15 (CH), 52.42 (2 CH_2_), 50.23 (CH_2_), 32.78 (2 CH_2_). IR (KBr) ν (cm^−1^) = 2936, 2828, 2092, 1510, 1447, 1300, 1239, 1027, 993, 908, 740. MS (EI) *m/z*: 311 (M^+^, 0.3), 219 (1), 189 (24), 172 (43), 146 (20), 136 (14), 121 (74), 91 (100). HRMS (EI) calcd. for C_20_H_27_N_2_O (M^+^ + H): 311.2123. Found: 311.2117.

*N*-Isobutyl-1-phenethylpiperidin-4-amine (**3a**): The compound was prepared according to general procedure 1 from 915 mg (4.5 mmol) 1-(2-phenylethyl)-4-piperidone, 4.67 mg (6.4 mmol) isobutylamine and 1.50 g (6.8 mmol) sodium triacetoxyborohydride to give 696 mg (59%) of **3a** as a colourless oil. ^1^H-NMR (500 MHz, chloroform-*d*) δ 7.30–7.26 (m, 2 H, 2 arom. CH), 7.22–7.17 (m, 3 H, 3 arom. CH), 3.00–2.93 (m, 2 H, CH_2_), 2.83–2.77 (m, 2 H, CH_2_), 2.60–2.54 (m, 2 H, CH_2_), 2.48–2.39 (m, 1 H, CH), 2.43 (d, *J* = 6.8 Hz, 2 H, CH_2_), 2.11–2.02 (m, 2 H, CH_2_), 1.93–1.86 (m, 2 H, CH_2_), 1.71 (dp, *J* = 13.3, 6.7 Hz, 1 H, CH), 1.46 (m, 2 H, CH_2_), 0.91 (d, *J* = 6.6 Hz, 6 H, 2 CH_3_). ^13^C-NMR (101 MHz, chloroform-*d*) δ 140.57 (quat. C), 128.71 (2 arom. CH), 128.35 (2 arom. CH), 125.97 (arom. CH), 60.68 (CH_2_), 54.99 (CH), 54.91 (2 CH_2_), 52.64 (CH_2_), 33.90 (CH_2_), 32.91 (2 CH_2_), 28.58 (CH), 20.76 (2 CH_3_). IR (KBr) ν (cm^−1^) = 2947, 2804, 1497, 1468, 1373, 1244, 1120, 1031, 978, 771, 744. MS (EI) *m/z*: 260 (M^+^, 0.2), 169 (100), 126 (48), 70 (93). HRMS (EI) calcd. for C_17_H_28_N_2_ (M^+^): 260.2253. Found: 260.2231. 

*N-Dodecyl-1-phenethylpiperidin-4-amine* (**3b**): The compound was prepared according to general procedure 1 from 610 mg (3.0 mmol) 1-(2-phenylethyl)-4-piperidone, 872 mg (4.5 mmol) *n*-dodecylamine and 1.339 g (6.0 mmol) sodium triacetoxyborohydride to give 677 mg (61%) of **3b** as a pale yellow oil. ^1^H-NMR (400 MHz, chloroform-*d*) δ 7.31–7.26 (m, 2 H, 2 arom. CH), 7.23–7.16 (m, 3 H, 3 arom. CH), 3.01–2.91 (m, 2 H, 2 CH_2_), 2.87–2.74 (m, 2 H, CH_2_), 2.66–2.54 (m, 4 H, 2 CH_2_), 2.46 (tt, *J* = 10.4, 3.9 Hz, 1 H, CH), 2.07 (td, *J* = 11.6, 2.3 Hz, 2 H, 2 CH_2_), 1.95–1.84 (m, 2 H, 2 CH_2_), 1.55–1.43 (m, 2 H, 2 CH_2_), 1.42–1.34 (m, 2 H, CH_2_), 1.34–1.20 (m, 18 H, 9 CH_2_), 0.87 (t, *J* = 6.7 Hz, 3 H, CH_3_). ^13^C-NMR (100 MHz, chloroform-*d*) δ 140.59 (quat. C), 128.71 (2 arom. CH), 128.35 (2 arom. CH), 125.96 (arom. CH), 60.70 (CH_2_), 55.06 (CH), 52.69 (2 CH_2_), 46.95 (CH_2_), 33.92 (CH_2_), 32.97 (CH_2_), 31.93 (2 CH_2_), 30.54 (CH_2_), 29.68 (CH_2_), 29.65 (CH_2_), 29.63 (2 CH_2_), 29.59 (CH_2_), 29.36 (CH_2_), 27.47 (CH_2_), 22.70 (CH_2_), 14.13 (CH_3_). IR (KBr) ν (cm^−1^) = 2921, 2849, 2805, 1466, 1454, 1371, 1122, 746, 697. MS (EI) *m/z*: 351 (M^+^ + H, 30), 259 (67), 216 (45), 147 (97), 117 (33), 96 (100), HRMS: calcd. for C_25_H_43_N_2_ (M^+^-H): 371.3426. Found: 371.3418.

*N-(4-(tert-Butyl)benzyl)-1-phenethylpiperidin-4-amine* (**3c**): The compound was prepared according to general procedure 1 from 610 mg (3.0 mmol) 1-(2-phenylethyl)-4-piperidone, 734 mg (4.5 mmol) 4-*tert*-butylbenzylamine and 1.339 g (6.0 mmol) sodium triacetoxyborohydride to give 609 mg (58%) of **3c** as a pale yellow oil. ^1^H-NMR (400 MHz, dichloromethane-*d*_2_) δ 7.33 (d, *J* = 8.4 Hz, 2 H, 2 arom. CH), 7.30–7.14 (m, 7 H, 7 arom. CH)), 3.75 (s, 2 H, CH_2_), 2.92 (dd, *J* = 11.8, 4.4 Hz, 2 H, 2 CH_2_), 2.76 (dd, *J* = 9.4, 6.4 Hz, 2 H, 2 CH_2_), 2.58–2.45 (m, 3 H, CH_2_, CH), 2.05 (td, *J* = 11.5, 2.2 Hz, 2 H, CH_2_), 1.94–1.83 (m, 2 H, 2 CH_2_), 1.44–1.33 (m, 2 H, 2 CH_2_), 1.30 (s, 9 H, 3 CH_3_). ^13^C-NMR (101 MHz, dichloromethane-*d*_2_) δ 150.92 (quat. C), 142.28 (quat. C), 139.53 (quat. C), 130.01 (2 arom. CH), 129.52 (2 arom. CH), 128.98 (2 arom. CH), 127.09 (arom. CH), 126.45 (2 arom. CH), 61.78 (CH_2_), 55.71 (CH), 53.72 (CH_2_), 51.67 (2 CH_2_), 35.61 (quat. C), 35.03 (CH_2_), 34.16 (2 CH_2_), 32.44 (3 CH_3_). MS (EI) *m/z*: 351 (M^+^+H, 30), 259 (67), 216 (45), 202 (12), 147 (97), 119 (27), 117 (27), 105 (36), 96 (100). HRMS (EI) calcd. for C_24_H_35_N_2_ (M^+^ + H): 351.2795. Found: 351.2793

*(S)-1-Phenethyl-N-(1-phenethyl)piperidin-4-amine* (**3d**): The compound was prepared according to general procedure 1 from 610 mg (3.0 mmol) 1-(2-phenylethyl)-4-piperidone, 610 (4.5 mmol) (*S*)-1-phenylethylamine and 1.34 g (6.0 mmol) sodium triacetoxyborohydride to give 748 mg (81%) of **3d** as a pale yellow oil. ^1^H-NMR (400 MHz, chloroform-*d*) δ 7.36–7.11 (m, 10 H, 10 arom. CH), 3.96 (q, *J* = 6.6 Hz, 1 H, CH), 2.96–2.85 (m, 2 H, 2 CH_2_), 2.81–2.73 (m, 2 H, CH_2_), 2.56–2.48 (m, 2 H, CH_2_), 2.38–2.27 (m, 1 H, CH), 2.02–1.89 (m, 3 H, 3 CH_2_), 1.78–1.68 (m, 1 H, CH_2_), 1.46–1.35 (m, 2 H, 2 CH_2_), 1.33 (d, *J* = 6.6 Hz, 3 H, CH_3_). ^13^C-NMR (100 MHz, chloroform-*d*) δ 146.18 (quat. C), 140.56 (quat. C), 128.69 (2 arom. CH), 128.40 (2 arom. CH), 128.34 (2 arom. CH), 126.78 (arom. CH), 126.52 (2 arom. CH), 125.96 (arom. CH), 60.66 (CH_2_), 54.52 (CH), 52.72 (CH_2_), 52.54 (CH_2_), 51.94 (CH), 33.89 (CH_2_), 33.69 (CH_2_), 32.62 (CH_2_), 25.12 (CH_3_). IR (KBr) ν (cm^−1^) = 2931, 2820, 1602, 1493, 1450, 1369, 1122, 1111, 762, 750. HRMS (EI) calcd. for C_21_H_29_N_2_ (M^+^ + 1): 309.2331. Found: 309.2323. $\propto \frac{20}{D}$ = −52.2° (c = 2.745, CHCl_3_).

*(S)-N-(1-Cyclohexylethyl)-1-phenethylpiperidin-4-amine* (**3e**): The compound was prepared according to general procedure 1 from 610 mg (3.0 mmol) 1-(2-phenylethyl)-4-piperidone, 610 mg (4.5 mmol) 1-(*S*)-cyclohexylethylamine and 1.34 g (6.0 mmol) sodium triacetoxyborohydride to give 797 mg (85%) of **3e** as a pale yellow oil. ^1^H-NMR (400 MHz, chloroform-*d*) δ 7.33–7.15 (m, 5 H, arom. CH), 3.00–2.89 (m, 2 H, 2 CH_2_), 2.85–2.76 (m, 2 H, CH_2_), 2.62–2.45 (m, 4 H, 2 CH, 2 CH_2_), 2.07 (qd, *J* = 11.6, 11.1, 2.1 Hz, 2 H, 2 CH_2_), 1.93–1.80 (m, 2 H, 2 CH_2_), 1.79–1.57 (m, 5 H, CH, 4 CH_2_), 1.47–1.29 (m, 2 H, 2 CH_2_), 1.30–1.05 (m, 6 H, 6 CH_2_), 1.05–0.96 (m, 2 H, 2 CH_2_), 0.96 (d, *J* = 6.4 Hz, 3 H, CH_3_). ^13^C-NMR (100 MHz, chloroform-*d*) δ 140.60 (quat. C), 128.71 (2 arom. CH), 128.34 (2 arom. CH), 125.96 (arom. CH), 60.72 (CH_2_), 53.98 (CH), 52.83 (CH_2_), 52.71 (CH_2_), 51.96 (CH), 43.30 (CH_2_), 33.93 (CH_2_), 33.83 (CH_2_), 32.97 (CH_2_), 30.02 (CH_2_), 28.19 (CH_2_), 26.80 (CH_2_), 26.65 (CH_2_), 26.52 (CH_2_), 17.70 (CH_3_). IR (KBr) ν (cm^−1^) = 2922, 2849, 2800, 1496, 1449, 1372, 1241, 1116, 1030, 979, 746, 698. MS (EI) *m/z*: 315 (M^+^, 0.3), 223 (69), 186 (20), 180 (85), 166 (19), 160 (45), 105 (38), 98 (48), 96 (100), 70 (82). HRMS (EI) calcd. for C_21_H_35_N_2_ (M^+^ + 1): 315.2800. Found: 315.2792. 

*N-(4-Chlorophenethyl)-1-phenethylpiperidin-4-amine* (**3f**): The compound was prepared according to general procedure 1 from 610 mg (3.0 mmol) 1-(2-phenylethyl)-4-piperidone, 700 mg (4.5 mmol) 4-chlorophenylethylamine and 1.339 g (6.0 mmol) sodium triacetoxyborohydride to give 686 mg (67%) of **3f** as a pale yellow oil. ^1^H-NMR (400 MHz, chloroform-*d*) δ 7.36–7.08 (m, 9 H, 9 arom. CH), 3.00–2.91 (m, 2 H, 2 CH_2_), 2.88 (t, *J* = 7.2 Hz, 2 H, CH_2_), 2.83–2.71 (m, 4 H, 3 CH_2_), 2.62–2.54 (m, 2 H, CH_2_), 2.53–2.44 (m, 1 H, CH), 2.12–2.03 (m, 2 H, CH_2_), 1.93–1.82 (m, 2 H, 2 CH_2_), 1.46–1.32 (m, 2 H, 2 CH_2_). ^13^C-NMR (100 MHz, chloroform-*d*) δ 140.53 (quat. C), 138.56 (quat. C), 131.90 (quat. C), 130.02 (2 arom. CH), 128.70 (2 arom. CH), 128.55 (2 arom. CH), 128.36 (2 arom. CH), 125.98 (arom. CH), 60.64 (CH), 54.89 (CH), 52.55 (2 CH_2_), 47.95 (CH_2_), 36.07 (CH_2_), 33.90 (2 CH_2_), 32.82 (CH_2_). IR (KBr) ν (cm^−1^) = 2938, 2797, 1492, 1451, 1358, 1143, 1120, 1043, 1014, 812, 739, 699. MS (EI) *m/z*: 343 (M^+^+H, 0.2), 208 (72), 194 (12), 160 (22), 139 (60), 125 (31), 103 (39), 96 (100), 91 (22), 70 (38). HRMS (EI) calcd. for C_21_H_28_N_2_Cl (M^+^ + H): 343.1941. Found: 343.1936.

*N-Hexyl-1-phenethylpiperidin-4-amine* (**3g**): The compound was prepared according to general procedure 1 from 610 mg (3.0 mmol) 1-(2-phenylethyl)-4-piperidone, 455 mg (4.5 mmol) *n*-hexylamine and 1.34 g (6.0 mmol) sodium triacetoxyborohydride to give 553 mg (64%) of **3g** as a pale yellow oil. ^1^H-NMR (400 MHz, chloroform-*d*) δ 7.24–7.19 (m, 2 H, 2 arom. CH), 7.15–7.09 (m, 3 H, 2 arom. CH), 2.94–2.85 (m, 2 H, CH_2_), 2.77–2.69 (m, 2 H, CH_2_), 2.57- 2.47 (m, 4 H, CH_2_), 2.39 (tt, *J* = 10.5, 4.1 Hz, 1 H, CH), 2.00 (td, *J* = 11.6, 2.4 Hz, 2 H, CH_2_), 1.87–1.79 (m, 2 H, CH_2_), 1.45–1.16 (m, 10 H, 5 CH_2_), 0.82 (t, *J* = 6.6 Hz, 3 H, CH_3_). ^13^C-NMR (100 MHz, chloroform-*d*) δ 140.56 (quat. C), 128.71 (2 arom. CH), 128.35 (2 arom. CH), 125.97 (arom. CH), 60.68 (CH_2_), 55.05 (CH), 52.68 (2 CH_2_), 46.93 (CH_2_), 33.90 (CH_2_), 32.92 (2 CH_2_), 31.80 (CH_2_), 30.48 (CH_2_), 27.15 (CH_2_), 22.64 (CH_2_), 14.07 (CH_3_). IR (KBr) ν (cm^−1^) = 2926, 2853, 2802, 1467, 1455, 1120, 745. 

*tert-Butyl 4-((4-(tert-butyl)benzyl)amino)piperidine-1-carboxylate* (**4a**): The compound was prepared according to general procedure 1 from 450 mg (2.3 mmol) 1-Boc-4-piperidone, 553 mg (3.4 mmol) 4-*tert*-butylbenzylamine and 1.0 g (4.52 mmol) sodium triacetoxyborohydride to give 783 mg (100%) of **4a** as a white solid. Mp: 73–75 °C. ^1^H-NMR (400 MHz, chloroform-*d*, 323 K) δ 7.33 (d, *J* = 8.4 Hz, 2 H, 2 arom. CH), 7.23 (d, *J* = 8.4 Hz, 2 H, 2 arom. CH), 4.04–3.91 (m, 2 H, 2 CH_2_), 3.78 (s, 2 H, CH_2_N), 2.83 (ddd, *J* = 13.8, 11.3, 2.9 Hz, 2 H, 2 CH_2_), 2.69 (tt, *J* = 9.9, 3.9 Hz, 1 H, CH), 1.92–1.80 (m, 2 H, 2 CH_2_), 1.45 (s, 9 H, 3 CH_3_), 1.32 (s, 9 H, 3 CH_3_), 1.35–1.28 (m, 2 H, 2 CH_2_). ^13^C-NMR (100 MHz, chloroform-*d,* 323 K) δ 154.93 (CO), 149.97 (quat. C), 137.69 (quat. C), 127.76 (2 arom. CH), 125.38 (2 arom. CH), 79.36 (quat. C), 54.33 (CH), 50.59 (CH_2_), 42.60 (2 CH_2_), 34.50 (quat. C), 32.61 (2 CH_2_), 31.43 (3 CH_3_), 28.53 (3 CH_3_). MS (EI) *m/z*: 346 (M^+^, 2), 289 (45), 162 (100), 147 (80), 57 (56). HRMS (EI) calcd. for C_21_H_34_N_2_O_2_: 346.2620. Found: 346.2620.

*tert-Butyl 4-(dodecylamino)piperidine-1-carboxylate* (**4b**)**:** The compound was prepared according to general procedure 1 from 598 mg (3.0 mmol) 1-Boc-4-piperidone, 834 mg (4.5 mmol) *n*-dodecylamine and 1.34 g (6.0 mmol) sodium triacetoxyborohydride to give 1.0 g (90%) of **4b** as a colourless oil. ^1^H-NMR (400 MHz, chloroform-*d*, 323 K) δ 4.13–3.76 (m, 2 H, 2 CH_2_), 2.80 (ddd, *J* = 13.9, 12.9, 2.8 Hz, 2 H, CH_2_), 2.61 (t, *J* = 7.1 Hz, 2 H, CH_2_), 2.60–2.54 (m, 1 H, CH), 1.85–1.76 (m, 2 H, 2 CH_2_), 1.50–1.44 (m, 2 H, 2 CH_2_), 1.45 (s, 9 H, 3 CH_3_), 1.36–1.19 (m, 20 H, 10 CH_2_), 0.87 (t, 3 H, CH_3_). ^13^C-NMR (100 MHz, chloroform-*d*) δ 154.83 (CO), 79.24 (quat. C) 54.99 (CH), 46.88 (CH_2_), 42.51 (2 CH_2_), 32.69 (CH_2_), 31.85 (2 CH_2_), 30.51 (CH_2_), 29.59 (CH_2_), 29.56 (CH_2_) 29.54 (2 CH_2_), 29.51 (CH_2_), 29.26 (CH_2_), 28.43 (3 CH_3_), 27.39 (CH_2_), 22.59 (CH_2_), 13.95 (CH_3_). IR (ATR, HCl of **4b**) ν (cm^−1^) = 2920, 2851, 2714, 1683, 1471, 1425, 1365, 1242, 1166, 1141, 866, 772, 720. MS (EI) *m/z*: 368 (M^+^, 8), 311 (100), 127 (50), 57 (86). HRMS (EI) calcd. for C_22_H_44_O_2_N_2_ (M^+^): 368.3403. Found: 368.3394.

*tert-Butyl 4-(octylamino)piperidine-1-carboxylate* (**4c**): The compound was prepared according to general procedure 1 from 897 mg (4.5 mmol) 1-Boc-4-piperidone, 872 mg (6.75 mmol) *n*-octylamine and 2.0 g (9.0 mmol) sodium triacetoxyborohydride to give 1.1 g (78%) of **4c** as a colourless oil. ^1^H-NMR (400 MHz, chloroform-*d,* 323 K) δ 4.08–3.91 (m, 2 H, 2 CH_2_), 2.87–2.73 (m, 2 H, 2 CH_2_), 2.60 (t, *J* = 7.3 Hz, 2 H, CH_2_), 2.61–2.52 (m, 1 H, CH), 1.86–1.74 (m, 2 H, 2 CH_2_), 1.52–1.42 (m, 2 H, 2 CH_2_). 1.45 (s, 9 H, 3 CH_3_), 1.35–1.22 (m, 12 H, 6 CH_2_), 0.87 (t, *J* = 7.2 Hz, 3 H, CH_3_). ^13^C-NMR (101 MHz, chloroform-*d,* 323 K) δ 154.86 (CO), 79.35 (quat. C), 55.12 (CH), 46.92 (CH_2_), 42.73 (2 CH_2_), 32.66 (2 CH_2_), 31.83 (CH_2_), 30.47 (CH_2_), 29.53 (CH_2_), 29.27 (CH_2_), 28.45 (3 CH_3_), 27.44 (CH_2_), 22.66 (CH_2_), 14.16 (CH_3_). MS (EI) *m/z*: 312 (M^+^, 0.8), 255 (6), 143 (2), 127 (4), 113 (3), 83 (13), 41 (100). HRMS (EI) calcd. for C_18_H_36_N_2_O_2_: 312.2777. Found: 312.2771.

*tert-Butyl 4-(cycloheptylamino)piperidine-1-carboxylate* (**4d**): The compound was prepared according to general procedure 1 from 598 mg (3.0 mmol) 1-Boc-4-piperidone, 509 mg (4.5 mmol) cycloheptylamine and 1.34 g (6.0 mmol) sodium triacetoxyborohydride to give 870 mg (98%) of **4d** as a colourless oil. ^1^H-NMR (400 MHz, chloroform-*d,* 323 K) δ 4.08–3.93 (m, 2 H, 2 CH_2_), 2.87–2.71 (m, 3 H, CH, 2 CH_2_), 2.72–2.60 (m, 1 H, CH), 1.87–1.73 (m, 4 H, 4 CH_2_), 1.72–1.08 (m, 12 H, 6 CH_2_), 1.45 (s, 9 H, 3 CH_3_). ^13^C-NMR (100 MHz, chloroform-*d*) δ 154.92 (CO), 79.35 (quat. C), 55.22 (CH), 51.95 (CH), 42.97 (2 CH_2_), 35.71 (2 CH_2_), 33.35 (2 CH_2_), 28.54 (3 CH_3_), 28.28 (2 CH_2_), 24.50 (2 CH_2_). IR (ATR) ν (cm^−1^) = 2980, 2921, 2852, 1688, 1419, 1364, 1234, 1169, 869, 769. MS (EI) *m/z*: 296 (M^+^, 20), 239 (6), 183 (100), 143 (54), 127 (44), 57 (54). HRMS (EI) calcd. for C_17_H_32_N_2_O_2_ (M^+^): 296.2464. Found: 296.2457.

*tert-Butyl 4-((pyridin-3-ylmethyl)amino)piperidine-1-carboxylate* (**4e**): The compound was prepared according to general procedure 1 from 1.1 g (6.2 mmol) 1-Boc-4-piperidone, 977 mg (9.03 mmol) 3-picolylamine and 2.67 g (12.0 mmol) sodium triacetoxyborohydride to give 1.75 g (100%) of **4e** as a colourless oil. ^1^H-NMR (400 MHz, chloroform-*d,* 323 K) δ 8.56 (d, *J* = 1.7 Hz, 1 H, arom. CH), 8.49 (dd, *J* = 4.8, 1.6 Hz, 1 H, arom. CH), 7.67 (dt, *J* = 7.8, 1.7 Hz, 1 H, arom. CH), 7.24 (ddd, *J* = 7.8, 4.8, 0.7 Hz, 1 H, arom. CH), 4.07–3.91 (m, 2 H, 2 CH_2_), 3.84 (s, 2 H, CH_2_N), 2.92–2.75 (m, 2 H, 2 CH_2_), 2.67 (ddd, *J* = 10.0, 6.0, 4.0 Hz, 1 H, CH), 1.92–1.78 (m, 2 H, 2 CH_2_), 1.64–1.47 (m, 2 H, 2 CH_2_), 1.45 (s, 9 H, 3 CH_3_). ^13^C-NMR (100 MHz, chloroform-*d,* 323 K) δ 154.88 (quat. C), 149.70 (arom. CH), 148.59 (arom. CH), 136.04 (quat. C), 135.66 (arom. CH), 123.39 (arom. CH), 79.48 (quat. C), 54.44 (CH), 48.27 (CH_2_), 42.55 (2 CH_2_), 32.60 (2 CH_2_), 28.53 (3 CH_3_). IR (KBr) ν (cm^−1^) = 2937, 2796, 1683, 1452, 1421, 1360, 1172, 1121, 1043, 843, 738, 697. MS (EI) *m/z*: 291 (M^+^, 26), 218 (18), 107 (23), 94 (40), 92 (47), 57 (100). HRMS: calcd. for C_16_H_25_N_3_O_2_: 291.1947. Found: 291.1942.

*tert-Butyl 4-((2-(pyrrolidin-1-yl)ethyl)amino)piperidine-1-carboxylate* (**4f**): The compound was prepared according to general procedure 1 from 598 mg (3.0 mmol) 1-Boc-4-piperidone, 977 mg (9.03 mmol) 1-pyrrolidineethanamine and 1.34 g (6.0 mmol) sodium triacetoxyborohydride to give 580 mg (65%) of **4f** as a colourless oil. ^1^H-NMR (400 MHz, chloroform-*d*) δ 4.08–3.93 (m, 2 H, 2 CH_2_), 2.89–2.73 (m, 4 H, 2 CH_2_), 2.67–2.55 (m, 7 H, CH, 3 CH_2_), 1.88–1.72 (m, 6 H, 3 CH_2_), 1.45 (s, 9 H, 3 CH_3_), 1.37–1.20 (m, 2 H, CH_2_). ^13^C-NMR (100 MHz, chloroform-*d*) δ 154.92 (CO), 79.39 (quat. C), 56.23 (2 CH_2_), 55.24 (CH), 54.23 (CH_2_), 45.30 (CH_2_), 42.68 (2 CH_2_), 32.61 (2 CH_2_), 28.53 (3 CH_3_), 23.59 (2 CH_2_). IR (KBr) ν (cm^−1^) = 3266, 2791, 1683, 1419, 1364, 1240, 1168, 1139, 873. MS (EI) *m/z*: 291 (M^+^, 2), 224 (6), 84 (100). HRMS: calcd. for C_16_H_31_N_3_O_2_: 297.2416. Found: 297.2411.

*N-(4-(tert-Butyl)benzyl)piperidin-4-amine* (**5a**): The compound was prepared according to general procedure 2 from 200 mg (0.58 mmol) of **4a** to give 120 mg (84%) of **5a** as a colourless oil. ^1^H-NMR (400 MHz, chloroform-*d*) δ 7.35 (d, *J* = 8.4 Hz, 2 H, 2 arom. CH), 7.25 (d, *J* = 8.4 Hz, 2 H, 2 arom. CH), 3.78 (s, 2 H, CH_2_), 3.15 (dt, *J* = 12.7, 3.7 Hz, 2 H, 2 CH_2_), 2.77–2.53 (m, 3 H, 2 CH_2_, CH), 2.00–1.87 (m, 2 H, 2 CH_2_), 1.42–1.23 (m, 2 H, 2 CH_2_), 1.31 (s, 9 H, 3 CH_3_). ^13^C-NMR (100 MHz, chloroform-*d*) δ 149.89 (quat. C), 137.38 (quat. C), 127.78 (2 arom. CH), 125.38 (2 arom. CH), 53.83 (CH), 50.26 (CH_2_), 44.66 (2 CH_2_), 34.47 (quat. C), 33.11 (2 CH_2_), 31.39 (3 CH_3_). IR (ATR) ν (cm^−1^) = 2954, 2863, 1461, 1363, 1269, 1201, 1128, 830, 800, 720.

*N-Dodecylpiperidin-4-amine* (**5b**): The compound was prepared according to general procedure 2, from 200 mg (0.54 mmol) of **4b** to give 120 mg (83%) of **5b** as a colourless oil. ^1^H-NMR (400 MHz, methanol-*d*_4_) δ 4.03–3.89 (m, 2 H, 2 CH_2_), 3.07–2.97 (m, 1 H, CH), 2.77–2.42 (m, 6 H, 3 CH_2_), 1.87–1.69 (m, 2 H, 2 CH_2_), 1.47–1.37 (m, 2 H, 2 CH_2_), 1.31–1.07 (m, 20 H, 10 CH_2_), 0.79 (t, *J* = 6.8 Hz, 3 H, CH_3_). ^13^C-NMR (100 MHz, methanol-*d*_4_) δ 54.81 (CH_2_), 46.03 (2 CH_2_), 45.67 (CH), 31.67 (CH_2_), 31.19 (CH_2_), 29.36 (CH_2_), 29.34 (CH_2_), 29.30 (CH_2_), 29.27 (CH_2_), 29.24 (CH_2_), 29.08 (CH_2_), 27.26 (CH_2_), 27.04 (CH_2_), 26.98 (CH_2_), 22.33 (CH_2_), 13.03 (CH_3_). IR (ATR, HCl) ν (cm^−1^) = 2920, 2851, 2721, 1471, 1426, 1243, 1167, 1142, 866, 772, 720. HRMS (EI) calcd. for C_17_H_35_N_2_ (M^+^-H): 267.8000. Found: 267.2795.

*N-Octylpiperidin-4-amine* (**5c**): The compound was prepared according to general procedure 2 from 200 mg (0.64 mmol) of **4c** to give 120 mg (88%) of **5c** as a colourless oil. ^1^H-NMR (400 MHz, methanol-*d*_4_) δ 3.58–3.39 (m, 3 H, 2 CH_2_, CH), 3.11 (td, *J* = 13.2, 2.7 Hz, 2 H, 2 CH_2_), 3.05–2.96 (m, 2 H, CH_2_), 2.40–2.28 (m, 2 H, 2 CH_2_), 2.07 (ddt, *J* = 22.3, 13.8, 7.1 Hz, 2 H, 2 CH_2_), 1.80–1.67 (m, 2 H, CH_2_), 1.47–1.24 (m, 10 H, 5 CH_2_), 0.89 (t, *J* = 6.8 Hz, 3 H, CH_3_). ^13^C-NMR (100 MHz, methanol-*d*_4_) δ 52.14 (CH), 44.94 (CH_2_), 42.18 (2 CH_2_), 31.55 (2 CH_2_), 28.87 (2 CH_2_), 26.38 (CH_2_), 26.10 (CH_2_), 24.91 (CH_2_), 22.39 (CH_2_), 13.34 (CH_3_). IR (KBr) ν (cm^−1^) = 2928, 1431, 1200, 1174, 1130, 836, 797, 721. MS (EI) *m/z*: 211 (M^+^-1, 46), 168 (46), 157 (38), 84 (74), 57 (100). HRMS (EI) calcd. for C_13_H_27_N_2_ (M^+^-H): 211.2180. Found: 211.2169.

*N-(Pyridin-3-ylmethyl)piperidin-4-amine* (**5e**): The compound was prepared according to general procedure 2 from 670 mg (2.3 mmol) of **4e** to give 100 mg (20%) of **5e** as a brown oil. ^1^H-NMR (400 MHz, methanol-*d*_4_) δ 8.55 (s, 1 H, NH), 8.51–8.40 (m, 2 H, NH, arom. CH), 7.91–7.84 (m, 1 H, arom. CH), 7.83–7.73 (m, 1 H, arom. CH), 7.45–7.38 (m, 1 H, arom. CH), 3.86 (s, 2 H, CH_2_), 3.45–3.36 (m, 2 H, 2 CH_2_), 3.05–2.95 (m, 2 H, 2 CH_2_), 2.91–2.80 (m, 1 H, CH), 1.71–1.55 (m, 2 H, 2 CH_2_), 1.38–1.24 (m, 2 H, 2 CH_2_).

*N-(2-(Pyrrolidin-1-yl)ethyl)piperidin-4-amine* (**5f**): The compound was prepared according to general procedure 2 from 200 mg (0.67 mmol) of **4e** to give 100 mg (76%) of **5e** as a colourless oil. ^1^H-NMR (400 MHz, chloroform-*d*) δ 3.25–3.11 (m, 4 H, 2 CH_2_), 2.76 (t, *J* = 6.3 Hz, 2 H, CH_2_), 2.72–2.64 (m, 1 H, CH), 2.61 (t, *J* = 6.3 Hz, 2 H, CH_2_), 2.55–2.47 (m, 4 H, 2 CH_2_), 2.02–1.87 (m, 2 H, 2 CH_2_), 1.82–1.72 (m, 4 H, 2 CH_2_), 1.43–1.25 (m, 2 H, 2 CH_2_). ^13^C-NMR (100 MHz, chloroform-*d*) δ 55.99 (2 CH_2_), 54.68 (CH), 54.13 (CH_2_), 45.18 (CH_2_), 44.65 (2 CH_2_), 32.97 (2 CH_2_), 23.43 (2 CH_2_). IR (KBr) ν (cm^−1^) = 2955, 2912, 2827, 1448, 1410, 1200, 1173, 1128, 799. MS (EI) *m/z*: 194 (2, M^+^-3), 113 (14), 97 (10), 84 (100). HRMS (EI) calcd. for C_11_H_21_N_3_ (M^+^-2H): 195.1736. Found: 195.1729.

*N-Dodecyl-N-(1-phenethylpiperidin-4-yl)acetamide* (**6a**): The compound was prepared according to general procedure 3 from 261 mg (0.7 mmol) of **3b** and 110 mg (2.0 mmol) acetyl chloride to give 280 mg (97%) of **6a** as a colourless oil. ^1^H-NMR (400 MHz, chloroform-*d*) δ 7.33–7.25 (m, 2 H, 2 arom. CH), 7.23–7.14 (m, 3 H, 3 arom. CH), 4.47–4.35 (m, 1 H, CH), 3.25–3.11 (m, 2 H, CH_2_), 3.10–2.98 (m, 2 H, CH_2_), 2.84–2.75 (m, 2 H, CH_2_), 2.65–2.56 (m, 2 H, CH_2_), 2.09 (s, 3 H, CH_3_), 1.76–1.63 (m, 3 H, 2 CH_2_), 1.61–1.47 (m, 3 H, 2 CH_2_), 1.37–1.18 (m, 20 H, 10 CH_2_), 0.88 (t, *J* = 6.8 Hz, 3 H, CH_3_). ^13^C-NMR (100 MHz, DMSO-*d*_6_) δ 169.67 (CO), 140.77 (quart. C), 128.99 (2 arom.), 128.63 (2 arom. CH), 126.23 (arom. CH), 60.09 (CH_2_), 56.05 (CH), 53.25 (2 CH_2_), 44.64 (CH_2_), 33.49 (CH_2_), 31.78 (CH_2_), 31.27 (CH_2_), 30.77 (CH_2_), 29.81 (CH_2_), 29.49 (CH_2_), 29.42 (2 CH_2_), 29.19 (CH_2_), 27.20 (CH_2_), 26.91 (2 CH_2_), 22.58 (CH_2_), 22.31 (CH_3_), 14.40 (CH_3_). IR (KBr) ν (cm^−1^) = 2923, 2852, 2804, 1644, 1455, 1420, 1369, 1287, 1122, 1041, 747, 699. MS (EI) *m/z*: 413 (M^+^-H, 0.1), 0.323 (100), 280 (14), 238 (15). HRMS (EI) calcd. for C_27_H_45_N_2_O (M^+^-H): 413.3532. Found: 413.3521.

*N-(4-(tert-Butyl)benzyl)-N-(1-phenethylpiperidin-4-yl)undec-10-enamide* (**6b**): The compound was prepared according to general procedure 3 from 501 mg (1.43 mmol) of **3c** and 580 mg (2.9 mmol) 10-undecenoyl chloride to give 575 mg (78%) of **6b** as a pale yellow oil. ^1^H-NMR (400 MHz, chloroform-*d*) δ 7.39–7.00 (m, 9 H, 9 arom. CH), 5.80 (ddt, *J* = 16.9, 10.1, 6.6 Hz, 1 H, -CH=), 5.04–4.87 (m, 2 H, CH_2_), 4.69–4.58 (m, 1 H, CH), 4.50 (s, 2 H, CH_2_), 3.09–2.95 (m, 2 H, 2 CH_2_), 2.80–2.71 (m, 2 H, CH_2_), 2.61–2.50 (m, 2 H, CH_2_), 2,24 (t, J = 7.4 Hz, 2 H, CH_2_), 2.16–1.98 (m, 4 H, 3 CH_2_), 1.71–1.53 (m, 6 H, 3 CH_2_), 1.43–1.18 (m, 10 H, 5 CH_2_), 1.31 (s, 9 H, 3 CH_3_). ^13^C-NMR (100 MHz, chloroform-*d*) δ 174.22 (CO), 150.02 (quat. C), 140.27 (quat. C), 139.20 (-CH=), 135.49 (quat. C), 128.64 (2 arom. CH), 128.38 (2 arom. CH), 126.03 (arom. CH), 125.51 (2 arom. CH), 125.35 (2 arom. CH), 114.11 (=CH_2_), 60.45 (CH_2_), 53.29 (2 CH_2_), 53.13 (CH_2_), 51.69 (CH), 46.20 (CH_2_), 33.85 (quat. C), 33.79 (2 CH_2_), 31.37 (3 CH_3_), 29.74 (CH_2_), 29.40 (CH_2_), 29.34 (CH_2_), 29.32 (CH_2_), 29.08 (CH_2_), 28.90 (CH_2_), 25.49 (CH_2_). IR (KBr) ν (cm^−1^) = 2925, 2853, 2802, 1643, 1457, 1412, 1373, 1194, 1120, 1031, 993, 908, 820, 747, 699. MS (EI) *m/z*: 516 (M^+^, 2), 425 (100), 147 (90). HRMS (EI) calcd. for C_35_H_52_N_2_O: 516.4080. Found: 516.3837.

*N-Dodecyl-N-(1-phenethylpiperidin-4-yl)butyramide (***6c**): The compound was prepared according to general procedure 3 from 373 mg (1.0 mmol) of **3b** and 160 mg (1.5 mmol) butanoyl chloride to give 427 mg (96%) of **6c** as a pale yellow oil. ^1^H-NMR (400 MHz, chloroform-*d*) δ 7.33–7.27 (m, 2 H, arom. CH), 7.24–7.15 (m, 3 H, 3 arom. CH), 4.53–3.50 (m, 1 H, CH), 3.24–3.00 (m, 4 H, 3 CH_2_), 2.86–2.74 (m, 2 H, CH_2_), 2.66–2.53 (m, 2 H, CH_2_), 2.35–2.23 (m, 2 H, 2 CH_2_), 2.20–2.02 (m, 2 H, CH_2_), 1.96–1.79 (m, 1 H, CH_2_), 1.78–1.45 (m, 9 H, 5 CH_2_), 1.35–1.22 (m, 16 H, 8 CH_2_), 1.00–0.94 (m, 3 H, CH_3_), 0.91–0.85 (m, 3 H, CH_3_). IR (KBr) ν (cm^−1^) = 2924, 2853, 1642, 1456, 1420, 1372, 1288, 1241, 1122, 1032, 747, 699. MS (EI) *m/z*: 441 (M^+^-H, 0.2), 351 (100), 238 (14), 105 (12). HRMS (EI) calcd. for C_22_H_43_N_2_O (M^+^-C_7_H_7_ (Bn)): 351.3375. Found: 351.3387.

*N-Dodecyl-N-(1-phenethylpiperidin-4-yl)cinnamamide* (**6d**): The compound was prepared according to general procedure 3 from 373 mg (1.0 mmol) of **3b** and 250 mg (1.5 mmol) cinnamoyl chloride to give 440 mg (88%) of **6d** as a pale yellow oil. ^1^H-NMR (400 MHz, chloroform-*d*) δ 7.72 (d, *J* = 15.3 Hz, 1 H, -CH=), 7.52 (dd, *J* = 7.6, 1.6 Hz, 2 H, 2 arom. CH), 7.44–7.34 (m, 3 H, 3 arom. CH), 7.33–7.27 (m, 2 H, 2 arom. CH), 7.25–7.16 (m, 3 H, 3 arom. CH), 6.80 (d, *J* = 15.3 Hz, 1 H, -CH=), 4.69–4.52 (m, 1 H, CH), 3.38–3.27 (m, 2 H, CH_2_), 3.15–3.04 (m, 2 H, CH_2_), 2.87–2.79 (m, 2 H, CH_2_), 2.65–2.58 (m, 2 H, CH_2_), 2.24–2.15 (m, 1 H, CH_2_), 1.84–1.69 (m, 3 H, 2 CH_2_), 1.38–1.23 (m, 22 H, 11 CH_2_), 0.87 (t, *J* = 6.8 Hz, 3 H, CH_3_). MS (EI) *m/z*: 502 (M^+^, 0.3), 501 (0.4), 411 (96), 281 (24), 131 (100), 103 (18). HRMS (EI) calcd. for C_34_H_49_N_2_O (M^+^-H): 501.3845. Found: 501.3824.

*N-(1-Benzylpiperidin-4-yl)-N-dodecyl-[1,1′-biphenyl]-2-carboxamide* (**6e**): The compound was prepared according to general procedure 3 from 717 mg (2.0 mmol) of **3b** and 648 mg (3 mmol) 2-phenylbenzoyl chloride (prepared from 607 mg (3.0 mmol) biphenyl-2-carboxylic acid and 595 mg (5.0 mmol) thionyl chloride) to give 900 mg (78%) of **6e** as a pale yellow oil. ^1^H-NMR (400 MHz, DMSO-*d*_6_) δ 11.25–10.65 (m, 1 H, NH), 7.68–7.23 (m, 14 H, 14 arom. CH), 4.35–3.93 (m, 2 H, CH_2_), 3.38–2.85 (m, 7 H, CH, 3 CH_2_), 2.81–2.58 (m, 2 H, CH_2_), 2.37–1.82 (m, 2 H, CH_2_), 1.57–1.44 (m, 2 H, CH_2_), 1.38–1.02 (m, 18 H, 9 CH_2_), 0.88 (t, *J* = 6.9 Hz, 3 H, CH_3_). IR (KBr) ν (cm^−1^) = 2922, 2852, 1623, 1455, 1437, 1419, 1302, 1047, 777, 744, 700. MS (ESI) *m/z*: 539 (100, M^+^ + H). HRMS (ESI) calcd. for C_37_H_51_N_2_O^+^ (M^+^+H)^+^: 539.3996. Found: 539.3999.

*N-Dodecyl-N-(1-phenethylpiperidin-4-yl)propionamide* (**6f**): The compound was prepared according to general procedure 3 from 373 mg (1.0 mmol) of **3b** and 139 mg (1.5 mmol) propionyl chloride to give 420 mg (98%) of **6f** as a pale yellow oil. ^1^H-NMR (400 MHz, chloroform-*d*) δ 7.34–7.27 (m, 2 H, 2 arom. CH), 7.25–7.16 (m, 3 H, 3 arom. CH), 4.53–4.41 and 3.62–3.49 (m, 1 H, CH), 3.27–3.01 (m, 4 H, 3 CH_2_), 2.87–2.75 (m, 2 H, CH_2_), 2.66–2.52 (m, 2 H, CH_2_), 2.42–2.25 (m, 2 H, 2 CH_2_), 2.22–2.01 (m, 2 H, CH_2_), 1.88 (qd, *J* = 13.9, 4.8 Hz, 1 H, CH_2_), 1.79–1.63 (m, 3 H, 2 CH_2_), 1.59–1.46 (m, 2 H, CH_2_), 1.34–1.20 (m, 20 H, 10 CH_2_), 1.19–1.10 (m, 3 H, CH_3_), 0.88 (t, *J* = 6.3 Hz, 3 H, CH_3_). ^13^C-NMR (100 MHz, chloroform-*d*) δ 173.78 (CO), 140.12 (quat. C), 128.67 (2 arom. CH), 128.41 (2 arom. CH), 126.08 (arom. CH), 60.49 (CH_2_), 55.33 (CH), 53.32 (CH_2_), 53.23 (2 CH_2_), 43.53 (CH_2_), 33.87 (CH_2_), 33.80 (CH_2_), 31.92 (CH_2_), 31.68 (CH_2_), 30.89 (CH_2_), 29.96 (CH_2_), 29.63 (CH_2_), 27.43 (2 CH_2_), 27.22 (CH_2_), 26.94 (CH_2_), 26.74 (CH_2_), 22.70 (CH_2_), 14.13 (CH_3_), 9.84 (CH_3_). IR (KBr) ν (cm^−1^) = 2918, 2851, 2814, 1611, 1456, 1378, 1253, 1145, 1117, 857, 808, 748, 699. MS (EI) *m/z*: 411 (M^+^, 2), 337 (100), 238 (16), 100 (56). HRMS (EI) calcd. for C_28_H_48_N_2_O: 428.3767. Found: 428.3674.

*N,1-Dibenzyl-N-methylpiperidin-4-amine* (**7a**): The compound was prepared according general procedure 1 from 568 mg (3.0 mmol) 1-benzyl-4-piperidone, 556 mg (4.5 mmol) N-benzylmethylamine and 1.34 g (6.0 mmol) sodium triacetoxyborohydride to give 701 mg (79%) of **7a** as a colourless oil. ^1^H-NMR (400 MHz, chloroform-*d*) δ 7.34–7.28 (m, 8 H, 8 arom. CH), 7.27–7.20 (m, 2 H, 2 arom. CH), 3.57 (s, 2 H, CH_2_), 3.49 (s, 2 H, CH_2_), 3.04–2.86 (m, 2 H, 2 CH_2_), 2.44 (tt, *J* = 11.6, 3.9 Hz, 1 H, CH), 2.19 (s, 3 H, CH_3_), 1.96 (td, *J* = 11.7, 2.3 Hz, 2 H, 2 CH_2_), 1.84–1.74 (m, 2 H, 2 CH_2_), 1.68 (td, *J* = 11.7, 3.5 Hz, 2 H, 2 CH_2_). ^13^C-NMR (100 MHz, chloroform-*d*) δ 140.16 (quat. C), 138.54 (quat. C), 129.16 (2 arom. CH), 128.76 (2 arom. CH), 128.19 (2 arom. CH), 128.16 (2 arom. CH), 126.93 (arom. CH), 126.73 (arom. CH), 63.15 (CH_2_), 60.98 (CH), 57.98 (CH_2_), 53.38 (2 CH_2_), 37.79 (CH_3_), 27.91 (2 CH_2_). IR (KBr) ν (cm^−1^) = 2940, 2793, 1658, 1452, 1363, 1254, 1147, 1122, 1044, 1017, 970, 906, 880, 789, 735, 697. MS (EI) m/z: 203 (M^+^, 11), 173 (44), 172 (30), 146 (24), 91 (100). HRMS (EI) calcd. for C_20_H_27_N_2_ (M^+^ + H): 295.2174. Found: 295.2167.

*N-Benzyl-N-methyl-1-phenethylpiperidin-4-amine* (**7b**): The compound was prepared according to general procedure 1 from 610 mg (3.0 mmol) 1-(2-phenylethyl)-4-piperidone, 556 mg (4.5 mmol) *N*-benzylmethylamine and 1.34 g (6.0 mmol) sodium triacetoxyborohydride to give 711 mg (77%) of **7b** as a colourless oil. ^1^H-NMR (400 MHz, chloroform-*d*) δ 7.41–7.13 (m, 10 H, 10 arom. CH), 3.59 (s, 2 H, CH_2_), 3.17–3.04 (m, 2 H, 2 CH_2_), 2.86–2.75 (m, 2 H, CH_2_), 2.64–2.54 (m, 2 H, CH_2_), 2.50–2.41 (m, 1 H, CH), 2.21 (s, 3 H, CH_3_), 2.02 (td, *J* = 11.8, 2.4 Hz, 2 H, 2 CH_2_), 1.89–1.80 (m, 2 H, 2 CH_2_), 1.71 (qd, *J* = 12.3, 3.8 Hz, 2 H, 2 CH_2_). ^13^C-NMR (100 MHz, chloroform-*d*) δ 140.50 (quat. C), 140.12 (quat. C), 128.70 (2 arom. CH), 128.38 (2 arom. CH), 128.22 (2 arom. CH), 126.76 (arom. CH), 126.00 (arom. CH), 60.91 (CH), 60.69 (CH_2_), 57.94 (CH_2_), 53.49 (2 CH_2_), 37.79 (CH_3_), 33.97 (CH_2_), 27.89 (2 CH_2_). IR (KBr) ν (cm^−1^) = 2937, 2800, 1495, 1453, 1359, 1313, 1263, 1196, 1120, 1043, 843, 738, 697. MS (EI) m/z: 308 (M^+^, 3), 217 (100), 160 (33), 174 (22), 187 (12), 146 (11), 120 (12), 105 (31), 96 (90), 91 (69), 70 (74), 42 (30). HRMS (EI) calcd. for C_21_H_28_N_2_: 308.2252. Found: 308.2246. 

*N-Dodecyl-1-phenethyl-N-(3-phenylpropyl)piperidin-4-amine* (**8d**): 503 mg (1.0 mmol) of **6d** was dissolved in 20 mL dry THF and 380 mg (10.0 mmol) LiAlH_4_ was added at 20 °C. The suspension was stirred for 1 h and then refluxed for 4 h. After careful addition of 20 mL ice water, the mixture was extracted with ethyl acetate (3 × 20 mL). The combined organic layers were dried over Na_2_SO_4_ and the solvent was evaporated. The crude residue was purified by flash column chromatography (ethyl acetate:triethylamine 10:1) to give 185 mg (38%) of **8d** as a pale yellow oil. ^1^H-NMR (400 MHz, chloroform-*d*) δ 7.30–7.23 (m, 4 H, arom. CH), 7.22–7.13 (m, 6 H, 6 arom. CH), 3.11–2.96 (m, 2 H, CH_2_), 2.84–2.71 (m, 2 H, CH_2_), 2.66–2.39 (m, 7 H, CH, 4 CH_2_), 1.96 (dd, *J* = 11.5 Hz, *J* = 2.0 Hz, 2 H, 2 CH_2_), 1.80–1.66 (m, 3 H, 3 CH_2_), 1.64–1.51 (m, 2 H, CH_2_), 1.48–1.33 (m, 3 H, 2 CH_2_), 1.35–1.09 (m, 20 H, 10 CH_2_), 0.88 (t, *J* = 6.8 Hz, 3 H, CH_3_). ^13^C-NMR (100 MHz, chloroform-*d*) δ 142.65 (quat. C), 140.54 (quat. C), 128.71 (2 arom. CH), 128.39 (4 arom. CH), 128.28 (2 arom. CH), 126.00 (arom. CH), 125.63 (arom. CH), 60.78 (CH_2_), 58.70 (CH), 53.82 (2 CH_2_), 50.78 (CH_2_), 50.37 (CH_2_), 34.00 (CH_2_), 33.81 (CH_2_), 31.97 (CH_2_), 30.92 (CH_2_), 29.75 (CH_2_), 29.74 (CH_2_), 29.70 (CH_2_), 29.68 (CH_2_), 29.41 (CH_2_), 29.29 (CH_2_), 28.27 (2 CH_2_), 27.57 (CH_2_), 22.74 (CH_2_), 14.18 (CH_3_). IR (KBr) ν (cm^−1^) = 2920, 2852, 1680, 1539, 1455, 1199, 1139, 800, 724, 700. MS (EI) *m/z*: 489 (M^+^-1, 4), 399 (22), 187 (30), 160 (42), 96 (100). HRMS: calcd. for C_34_H_54_N_2_ (M^+^): 490.4287. Found: 490.4277.

*N-([1,1′-Biphenyl]-2-ylmethyl)-1-benzyl-N-dodecylpiperidin-4-amine* (**8e**): 349 mg (0.6 mmol) of **6e** as hydrochloride was dissolved in 20 mL dry THF and 224 mg (5.9 mmol) LiAlH_4_ was added at 20 °C. The suspension was stirred for 1 h and then refluxed for 4 h. After careful addition of 20 mL ice water, the mixture was extracted with ethyl acetate (3 × 20 mL). The combined organic layers were dried over Na_2_SO_4_ and the solvent was evaporated. The crude residue was purified by flash column chromatography (ethyl acetate/trimethylamine 10:1) to give 310 mg (100%) of **8e** as a colourless oil. ^1^H-NMR (400 MHz, chloroform-*d*) δ 7.68 (d, *J* = 8.2 Hz, 1 H, arom. CH), 7.40–7.20 (m, 12 H, 12 arom CH), 7.17 (dd, *J* = 7.5, 1.3 Hz, 1 H, arom. CH), 3.53 (s, 2 H, CH_2_), 3.44 (s, 2 H, CH_2_), 2.90–2.79 (m, 2 H, 2 CH_2_), 2.39–2.32 (m, 1 H, CH), 2.33 (t, *J* = 7.0 Hz, 2 H, CH_2_), 1.82 (td, *J* = 11.0, 4.0 Hz, 2 H, 2 CH_2_), 1.52–1.48 (m, 4 H, 3 CH_2_), 1.35–1.11 (m, 20 H, 10 CH_2_), 0.87 (t, *J* = 6.7 Hz, 3 H, CH_3_). ^13^C-NMR (100 MHz, chloroform-*d*) δ 141.88 (quat. C), 141.63 (quat. C), 138.75 (quat. C), 138.47 (quat. C), 129.62 (arom. CH), 129.33 (2 arom. CH), 129.25 (arom. CH), 129.18 (2 arom. CH), 128.12 (2 arom. CH), 127.86 (2 arom. CH), 127.14 (arom. CH), 126.89 (arom. CH), 126.71 (arom. CH), 125.99 (arom. CH), 63.18 (CH_2_), 57.56 (CH), 53.60 (2 CH_2_), 51.71 (CH_2_), 49.97 (CH_2_), 31.94 (CH_2_), 29.70 (CH_2_), 29.69 (2 CH_2_), 29.67 (2 CH_2_), 29.56 (CH_2_), 29.38 (CH_2_), 28.62 (CH_2_), 27.88 (CH_2_), 27.36 (CH_2_), 22.71 (CH_2_), 14.14 (CH_3_). IR (KBr) ν (cm^−1^) = 2922, 2851, 2799, 1454, 1365, 1147, 1072, 750, 699. MS (EI) *m/z*: 524 (M^+^, 1), 425 (23), 350 (64), 173 (89), 167 (100). HRMS: calcd. for C_37_H_52_N_2_ (M^+^): 524.4130. Found: 524.4127.

### 4.2. Biology

#### 4.2.1. Antifungal Susceptibility Testing

The model strain *Yarrowia lipolytica* (DSMZ Braunschweig, DSM No. 8218) culture was diluted with AC-Agar (Sigma Aldrich, Steinheim, Germany) to a McFarland standard of 0.5. Five microliters of this dilution was again diluted with AC-Agar to 20 mL, and 99 µL of this dilution was plated in each well of a 96-well plate. Then, 1 µL of 12 dilutions of the compounds from 20 mg/mL to 10 µg/mL were plated in triplicate in the 96-well plate and the plate was incubated at 28 °C for 48 h. After this, the growth of *Yarrowia lipolytica* was determined visually and MIC_100_ was defined as 100% growth inhibition.

Determination of MICs of clinically relevant species was carried out according to the European Committee of Antifungal Susceptibility Testing [23]. Clinical isolates belonging to the group of *Aspergilli*, *Candida* spp., or *Mucormycetes* were chosen from the strain collection of the Institute of Hygiene and Medical Microbiology, Innsbruck, Austria. Details on the strain set tested are given in Supporting Information Table S3. Reference strains: *Aspergillus flavus* (ATCC 204304), *Aspergillus fumigatus* (ATCC 204305), *Aspergillus terreus* (ATCC 3633), and *Candida krusei* (ATCC 6258), were included as control strains according to EUCAST protocol. All mold isolates were adjusted to an inoculum size of 2 × 10^5^ spores/mL, and spores were counted with a hemocytometer (Neubauer). Concentrations of *Candida cells* were determined using a photometer and adjusted according to McFarland standard of 0.5. For molds, MIC_90_, defined as 90% growth inhibition compared to controls without antifungal, was determined visually at 24 h; if growth in the control wells was not sufficient, then it was determined at 48 h (*A. terreus*). For yeasts, MIC was determined using a plate reader and defined as MIC_80_ (80% growth inhibition). All substances were dissolved in DMSO and diluted in RPMI 1640 medium, containing 2% glucose. 

As compounds **2b** and **3b** exhibited promising growth-inhibiting activity at low concentrations, minimal fungicidial concentrations (MFCs) were determined according to literature [36,37] against selected *Candida* and *Aspergillus* isolates. In brief, 100 µL of samples containing the MIC concentration, one dilution step lower than the MIC, and 1 and 2 dilution steps higher than the MIC were plated on Sabouraud agar plates and the number of CFUs was compared to the growth control (no antifungal drug). Amorolfin hydrochloride was used for comparison. Fungicidial and fungistatic activity were defined according to Warn et al. [36].

#### 4.2.2. Cytotoxicity Assays

HL-60 cells (human leukemia cells, DSM No. ACC3) were obtained from DSMZ (German Collection of Microorganisms and Cell Cultures, Braunschweig, Germany) and cultivated in RPMI 1640 medium with 10% fetal bovine serum (FBS), both from PAA Laboratories, Cölbe, Germany) without the addition of antibiotics at 37 °C in a humidified atmosphere containing 5% CO_2_. Human umbilical vein endothelial cells (HUVECs) were purchased from Promocell and cultivated with ECGM Kit Enhanced (PELO Biotech, Planegg, Germany), supplemented with 10% FBS and 1% penicillin/streptomycin/amphothericin B (all purchased from PAN Biotech, Aidenbach, Germany). MCF10A cells (healthy epithelial cell line) were purchased from ATCC and cultivated with DMEM/F-12 containing L-glutamine, 15 mM HEPES, and 1.2 g/L NaHCO_3_ (PAN Biotech, Aidenbach, Germany), supplemented with 5% horse serum, heat-inactivated, New Zealand origin (Gibco, Fisher Scientific, Waltham, MA, USA), 100 ng/mL epidermal growth factor (Peprotech, Cranbury, NJ, USA), 10 ng/mL insulin (Santa Cruz Biotechnology, Heidelberg, Germany), 1 mg/mL hydrocortisone (Sigma Aldrich, St. Louis, MO, USA), and 100 ng/mL cholera toxin (Sigma Aldrich, St. Louis, MO, USA). Cytotoxicity was assessed using MTT and CellTiter-Blue^®^ (CTB, Promega, Fitchburg, WI, USA) cell viability assays, both of which rely on determining the metabolic activity of living cells. HUVECs were cultured for a maximum of six passages. The MTT assay for cytotoxicity was performed with these cells as described by Horling et al. [38].

Cytotoxicity assays on HUVECs and MCF10A cells were carried out according to Schütz et al. [39]. HUVECs and MCF10A cells were seeded at a density of 5 × 10^3^ cells per well of a 96-well plate.

#### 4.2.3. Toxicity Assay in Galleria Mellonella

Per sample, 20 *Galleria mellonella* larvae, obtained from SA.GI.P. (Bagnacavallo, Italy), were injected at three different concentrations of amorolfine hydrochloride, **2b** or **3b**, diluted in PBS. The control larvae were injected with 20 µL of PBS or left untouched. Larvae were incubated at 37 °C and survival was monitored every 24 h over 4 days. The average survival rate of two independent experiments (40 larvae in total) was plotted in Kaplan Meier curves and statistical difference determined by log rank test (utilizing GraphPad Prism). The concentrations to be tested were selected based on the average MIC values determined for each drug. Applying 20 µL of 100 µM represents the MIC (4 µg/mL) of **2b** and **3b** per g larvae, 500 µM represents the MIC obtained for amorolfine hydrochloride (16 µg/mL), and additionally 10-fold the MIC of the novel substances was chosen.

#### 4.2.4. Identification of the Target Enzyme(s) in Ergosterol and Cholesterol Biosynthesis

All fungal strains were cultivated according to Müller et al. [3]. Three milligrams of dry fungal biomass was used for sterol extraction, as described by Müller et al. [3]. The results represent the mean of two independent biological samples. The following concentrations were used: *Aspergillus fumigatus* amorolfine hydrochloride (4.0 µg/mL; 15 µg/mL), **2b** (3.5 µg/mL; 8.0 µg/mL), **3b** (4.0 µg/mL); *Candida albicans* amorolfine hydrochloride (4.0 µg/mL; 15 µg/mL), **2b** (1.8 µg/mL; 4.0 µg/mL), **3b** (4.0 µg/mL); *Candida glabrata* amorolfine hydrochloride (7.5 µg/mL), **2b** (3.5 µg/mL; 7.9 µg/mL), **3b** (4.0 µg/mL). For all concentrations, similar results were obtained. Hence only one concentration is shown in Table 4.

Sterol pattern was determined by GC-MS, according to Müller et al. [3,40]. The quantification, managed with an external calibration with ergosterol, consists of six levels with concentrations up to 20 µg/mg. The base peak of each sterol TMS ether was taken as a quantifier ion for calculating the peak areas for internal standard cholestane *m/z* 217, ergosta-5,8,22-trien-3β-ol (lichesterol) *m/z* 363, ergosta-5,7,22-trien-3β-ol (ergosterol) *m/z* 363, ergosta-7,22-dien-3β-ol *m/z* 343, ergosta-7,22,24(28)-trien-3β-ol *m/z* 343, ergosta-5,7,24(28)-trien-3β-ol *m/z* 363, ergosta-5,7-dien-3β-ol of *m/z* 365, ergosta-7,24(28)-dien-3β-ol (episterol) *m/z* 343, 4,4,14-trimethylcholesta-8,24-dien-3β-ol (lanosterol) *m/z* 393, 4,4,14-trimethylergosta-8,24(28)-dien-3β-ol (eburicol) *m/z* 407, ergosta-8,14,24(28)-trien-3β-ol *m/z* 369, 4-methylergosta-8,24(28)-dien-3β-ol (4-methylfecosterol) *m/z* 379, ergosta-5,8,24(28)-trien-3β-ol *m/z* 363, ergosta-5,8,22,24(28)-tetraen-3β-ol *m/z* 466, ergosta-8,24(28)-dien-3β-ol *m/z* 365, ergosta-8,24(28)-diendi-3β,?-ol (hydroxyfecosterol) *m/z* 468 (according to ref. [41]), and ergosta-5,7,22,24(28)-tetraen-3β-ol *m/z* 361. The percentage of each sterol was determined and plotted against the percentage sterol content of the untreated sample (fold change). The target identification in distal cholesterol biosynthesis was performed according to Müller et al. [35].

## Figures and Tables

**Table 1:** Antifungal activity (MIC_100_ for *Yarrowia lipolytica*, MIC_90_ for molds, and MIC_80_ for yeasts) of six novel 4-aminopiperidine derivatives, amorolfine hydrochloride (**A**), and voriconazole (**V**) against the model strain *Y. lipolytica* and clinically relevant fungal species; () = tested fungal isolates in total.

Strain	MIC Values [µg/mL]								
	Compound	A	V	2b	2c	3b	4b	5b	6a
**Model strain**	*Y. lipolytica* (1)	0.6	0.4	2	25	0.8	3	5	75
***Aspergillus* spp.**	*A. flavus* (1)	>16	0.25	16	>16	4–8	16	16	>16
	*A. fumigatus* (2)	>16	0.125	8	>16	2–8	16	16	>16
	*A. terreus* (2)	8–16	0.125	4–8	8–16	1–2	8	4–8	4–8
***Candida* spp.**	*C. auris* (1)	16	>16	4	64	2–4	16	8	>16
	*C. glabrata* (1)	>16	>16	4	32	1–4	8–16	4–8	>16
	*C. krusei* (5)	1–>16	0.5–>16	2–4	2–64	1–4	1–16	4–8	>16
***Mucor-*** ***mycetes***	*A. elegans* (1)	16	>16	>16	>16	8	>16	>16	>16
	*L. corymbifera* (1)	16	>16	>16	>16	>16	>16	>16	16
	*L. ramosa* (1)	8	8	8	>16	8	16	16	>16
	*M. circinelloides* (1)	16	>16	16	>16	4	16	16	16
	*R. arrhizus* (1)	8	16	16	>16	8	>16	16	>16
	*R. microsporus* (2)	8	16	8	>16	8	16	8	16
	*S. racemosum* (1)	16	>16	>16	>16	>16	>16	>16	16

**Table 2:** Antifungal activity of amorolfine hydrochloride (**A**), **2b**, and **3b** against clinical isolates (*Aspergillus* and *Candida* species). MIC ranges represent the MIC_90_ values obtained for 6 *Aspergillus* isolates and the MIC_80_ values obtained for 6–7 isolates of each *Candida* species in all experiments. MFC ranges represent the MFCs determined for selected *Aspergillus* (1 per species) and *Candida* (1 isolate of *C. albicans* and *C. tropicalis* and 2 isolates of *C. glabrata* and *C. krusei*) isolates; () = tested fungal isolates in total. All experiments were carried out in duplicates.

Strain		MIC Ranges (µg/mL)			MFC Ranges (µg/mL)		
	Compound	A	2b	3b	A	2b	3b
***Aspergillus* spp.**	*A. flavus* (6)	>16	8–16	8–16	>16	16	16
	*A. fumigatus* (6)	>16	8–16	4–8	>16	16	16
	*A. terreus* (6)	>16	8–16	4–16	>16	16	16
***Candida* spp.**	*C. albicans* (6)	4–16	4–8	2–4	>16	8	4–8
	*C. glabrata* (7)	4–>16	4–8	1–4	>16	4–8	2–8
	*C. krusei* (6)	0.5–>16	2–4	1–4	16–>16	4–8	2–4
	*C. tropicalis* (6)	8–>16	4–8	2–4	>16	8	8

**Table 3:** Cytotoxic activity of selected compounds against human cell lines. Cell viability was determined by MTT assay (HL-60) and CTB assay (HUVEC, MCF10A); mean IC_50_ values (*n* = 3) for amorolfine hydrochloride (**A**), posaconazole (**P**), and voriconazole (**V**), and for the promising compounds **2b** and **3b** are shown.

Cell Line	IC_50_ (µM (µg/mL))					
	Compound	A	P	V	2b	3b
HL-60		17 (6.0)	5 (3.5)	>50 (>17.5)	7 (2.5)	4 (1.5)
HUVEC		50 (17.7)	14 (9.8)	>50 (>17.5)	5 (1.8)	5 (1.9)
MCF10A		>50 (>17.7)	22 (15.4)	>50 (>17.5)	6 (2.1)	7 (2.6)

**Table 4:** Identification of the target enzyme(s) in ergosterol biosynthesis. Heatmap of the detected sterols. The relative sterol amount has been normalized to that of untreated samples [33]. Red: marker sterols according to Müller et al. [3]; boxes in red colour > 10-fold change, in blue colour no changes were observed, and in purple colour <0.1-fold change. **A,** amorolfine hydrochloride; n.d., not detected; (), trivial name; ?, unknown position of the second hydroxy group. Test concentrations: *Aspergillus fumigatus*: amorolfine hydrochloride (4.0 µg/mL), **2b** (3.5 µg/mL), **3b** (4 µg/mL); *Candida albicans*: amorolfine (4.0 µg/mL), **2b** (3.5 µg/mL), **3b** (4.0 µg/mL); *Candida glabrata*: amorolfine hydrochloride (7.5 µg/mL), **2b** (3.5 µg/mL), **3b** (4.0 µg/mL).

Enzyme	Sterol	Strain	*A. fumigatus*			*C. flbicans*			*C. glabrata*		
		Compound	2b	3b	A	2b	3b	A	2b	3b	A
sterol C14- demethylase	14-methylergosta-8,24(28)-dien-3 β ,6 α -diol		n.d.			n.d.			n.d.		
	4,4,14-trimethylcholesta-8,24(28)-dien-3β-ol (lanosterol)										
	4,4,14-trimethylergosta-8,24(28)-dien-3β-ol (eburicol)										
sterol C14- reductase	4,4-dimethylcholesta-8,14,24-trien-3β-ol (FF-MAS)		n.d.			n.d.			n.d.		
	ergosta-8,14,24(28)-trien-3β-ol										
sterol C4-demethylase complex	4,4-dimethylcholesta-8,24-dien-3β-ol (T-MAS)		n.d.			n.d.			n.d.		
	4-methylergosta-8,24(28)-dien-3β-ol										
sterol C24-methyltransferase	cholesta-5,7,24-trien-3β-ol		n.d.			n.d.			n.d.		
	cholesta-5,7,22,24-tetraen-3β-ol		n.d.			n.d.			n.d.		
sterol C8- isomerase	ergosta-5,8,22-trien-3β-ol (lichesterol)										
	ergosta-5,8,24(28)-trien-3β-ol										
	ergosta-5,8,22,24(28)-tetraen-3β-ol										
	ergosta-8,24(28)-dien-3β-ol										
	ergosta-8,24(28)-diendi-3β,?-ol (hydroxyfecosterol)										
sterol C5- desaturase	ergosta-7,22-dien-3β-ol										
	ergosta-7,24(28)-dien-3β-ol (episterol)										
	ergosta-7,22,24(28)-trien-3β-ol										
sterol C22- desaturase	ergosta-5,7-dien-3β-ol										
	ergosta- 5,7,24(28)-trien-3β-ol										
sterol C24- reductase	ergosta-5,7,22,24(28)-tetraen-3β-ol		n.d.			n.d.			n.d.		
	ergosta-5,7,22-trien-3β-ol (ergosterol)										

**Table 5:** Identification of the target enzyme(s) of compounds **2b** and **3b** in cholesterol biosynthesis on HL-60 cells. Mainly accumulating sterols under incubation and identified target enzymes. (), trivial name; n.d., not determined [35].

Compound	Mainly Accumulating Sterol(s)	Inhibited Enzyme(s)
**2b**	0.1 µM: cholesta-8,14-dien-3β-ol 1 µM: cholesta-8,14-dien-3β-ol, cholesta-8-en-3β-ol (zymostenol), cholesta-5,7-dien-3β-ol (7-dehy drocholesterol), cholesta-7-en-3β-ol (lathosterol); 10 µM: toxic	0.1 µM: sterol C14-reductase, sterol C8-isomerase 1 µM: multi-enzyme inhibition 10 µM: n.d.
**3b**	0.1 µM: no accumulation; 1 µM: cholesta-8,14-dien-3β-ol, cholesta-8-en-3β-ol (zymostenol), cholesta-5,7-dien-3β-ol (7-dehydrocholesterol), cholesta-7-en-3β-ol (lathosterol); 10 µM: toxic	0.1 µM: n.d. 1 µM: multi-enzyme inhibition 10 µM: n.d.

## Data Availability

NMR data of the compounds are available from the authors upon request.

## Supplementary Materials

The following are available online: Supporting Information Table S1: Results of the antifungal activity screening for all compounds on *Yarrowia lipolytica;* Supporting Information Table S2: Results of the identification of the target enzyme(s) in cholesterol biosynthesis for compounds **2b**, **2c**, **2f**, **2g**, **3b**, **4b**, **4c**, **5b**, and **6b** on HL-60 cells; Supporting Information Table S3: Fungal strains used in this study; Supporting Information Figure S1: Exemplary antifungal activity of **2b**, **3b** and amorolfine (**A**) against the two major fungal pathogens, *A. fumigatus* and *C. albicans*.

## Sample Availability

Samples of the compounds are available from the authors.
