# Supplementary material for: Synthesis, Biological Evaluation, and Structure–Activity Relationships of 4-Aminopiperidines as Novel Antifungal Agents Targeting Ergosterol Biosynthesis
Source: Molecules. 2021 Nov 28;26(23):7208. doi: 10.3390/molecules26237208 (PMC8658910; doi:10.3390/molecules26237208)

Supporting Information Figure S1. Exemplary antifungal activity of **2b**, **3b** and amorolfine hydrochloride (**A**) against the two major fungal pathogens, *Aspergillus fumigatus* and *Candida albicans*. MFCs were determined after confrontation of fungal spores with antifungal substances for 24 h. Yellow boxes represent MIC values, while green boxes represent the MFCs determined for each substance

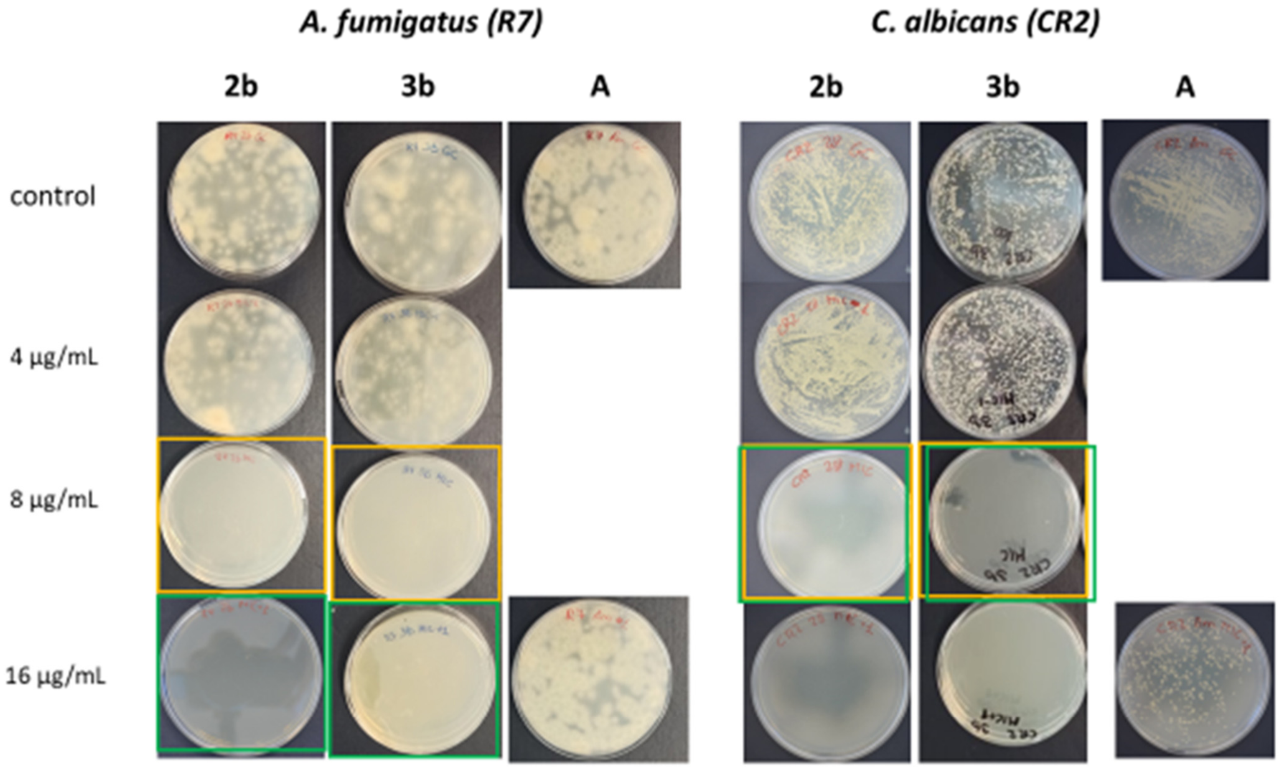

Supplement: Supplementary file 1 [file molecules-26-07208-s001.zip › Supporting Information Figure S1.pdf]
