# Supplementary material for: Synthesis, Biological Evaluation, and Structure–Activity Relationships of 4-Aminopiperidines as Novel Antifungal Agents Targeting Ergosterol Biosynthesis
Source: Molecules. 2021 Nov 28;26(23):7208. doi: 10.3390/molecules26237208 (PMC8658910; doi:10.3390/molecules26237208)
Supplement: Supplementary file 1 [file molecules-26-07208-s001.zip › Supporting Information Table S1.pdf]

Supporting Information Table S1. Overview of synthesized compounds and antifungal activity screening on *Yarrowia lipolytica*; n.d. not determined. Reference antifungals: amorolfine hydrochloride, terbinafine hydrochloride, posaconazole, voriconazole.

Name	Structure	Formula	Molecular weight [g/mol]	MIC [µg/mL]
**Amorolfine hydrochloride**	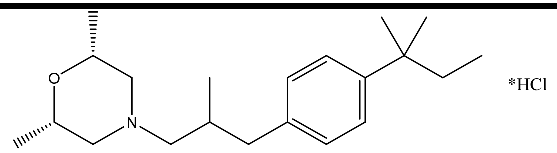	$C_{21}H_{35}NO$	317.52	0.6
**Terbinafine hydrochloride**	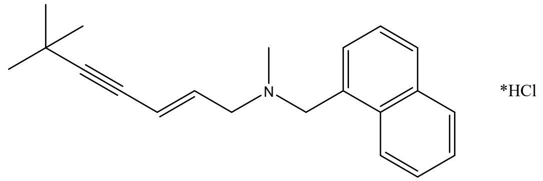	$C_{21}H_{25}N$	291.44	0.4
**Posaconazole**	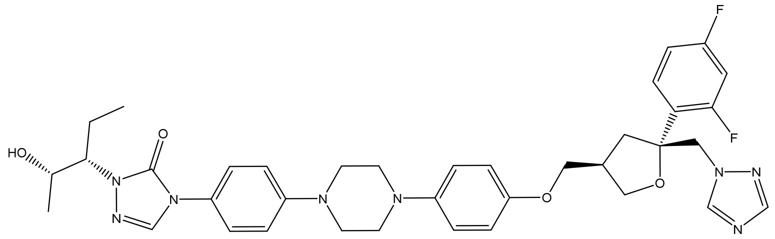	$C_{37}H_{42}F_2N_8O_4$	700.79	0.8
**Voriconazole**	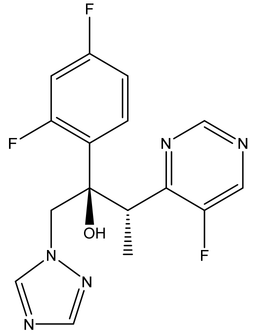	$C_{16}H_{14}F_3N_5O$	349.32	0.4

Name	Structure	Formula	Molecular weight [g/mol]	MIC [µg/mL]
**2a**	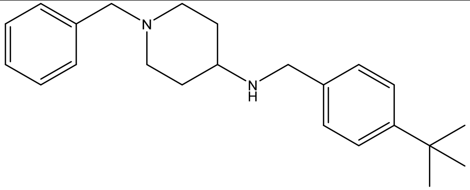	$C_{23}H_{32}N_2$	336.26	10
**2b**	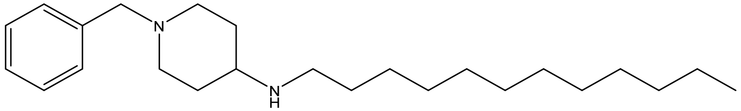	$C_{24}H_{42}N_2$	358.61	2
**2c**	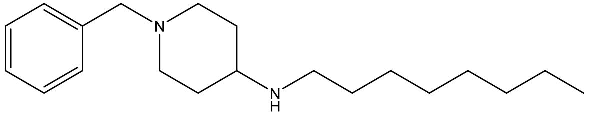	$C_{20}H_{34}N_2$	302.51	25
**2d**	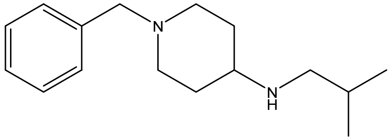	$C_{16}H_{26}N_2$	246.40	>200
**2e**	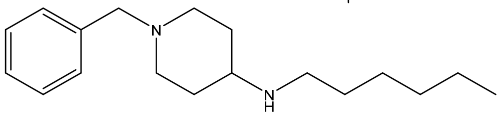	$C_{18}H_{30}N_2$	274.45	>200
**2f**	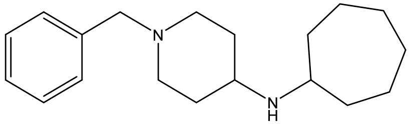	$C_{19}H_{30}N_2$	286.46	>200
**2g**	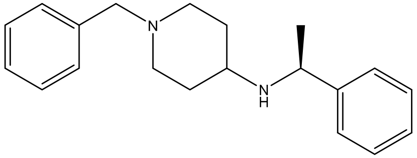	$C_{20}H_{26}N_2$	294.21	>200

Name	Structure	Formula	Molecular weight [g/mol]	MIC [µg/mL]
**2h**	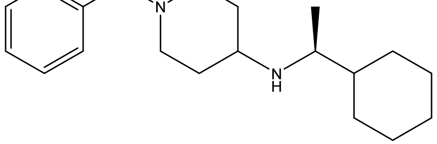	$C_{20}H_{32}N_2$	300.49	>200
**2i**	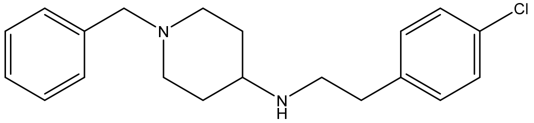	$C_{20}H_{25}ClN_2$	328.17	100
**2j**	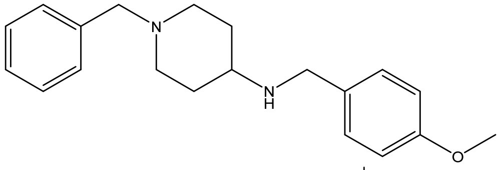	$C_{20}H_{26}N_2O$	310.44	>200
**3a**	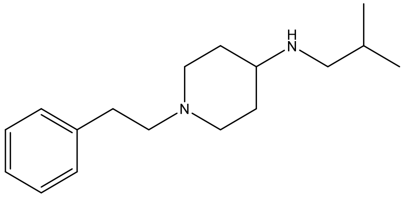	$C_{17}H_{28}N_2$	260.23	200
**3b**	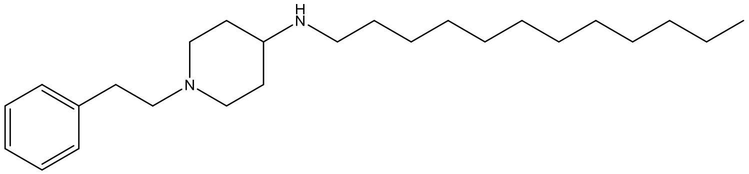	$C_{25}H_{44}N_2$	372.64	0.8
**3c**	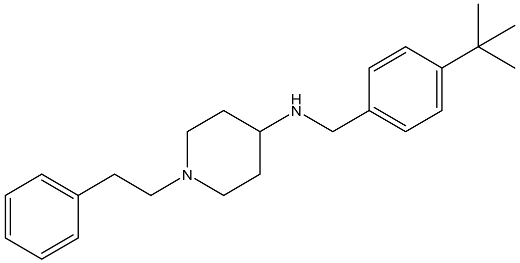	$C_{24}H_{34}N_2$	350.55	5

Name	Structure	Formula	Molecular weight [g/mol]	MIC [µg/mL]
**3d**	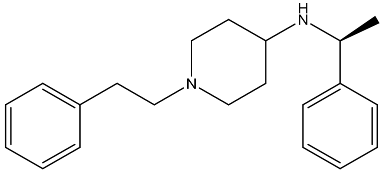	$C_{21}H_{28}N_2$	308.47	>200
**3e**	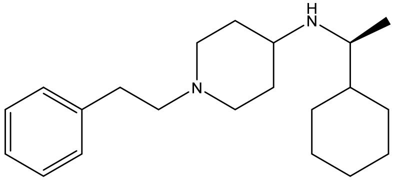	$C_{21}H_{34}N_2$	314.27	>200
**3f**	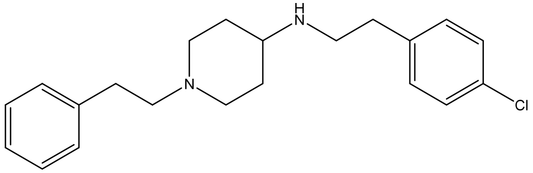	$C_{21}H_{27}ClN_2$	342.91	50
**3g**	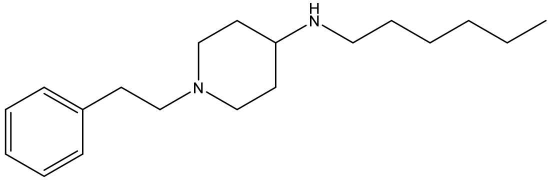	$C_{19}H_{32}N_2$	288.48	75
**4a**	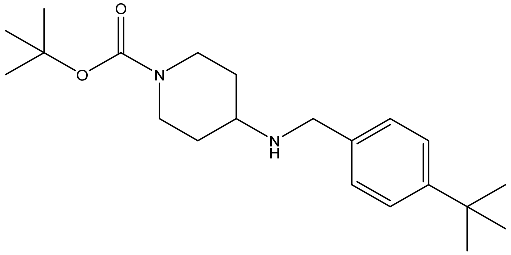	$C_{21}H_{34}N_2O_2$	346.26	25

Name	Structure	Formula	Molecular weight [g/mol]	MIC [μg/mL]
**4b**	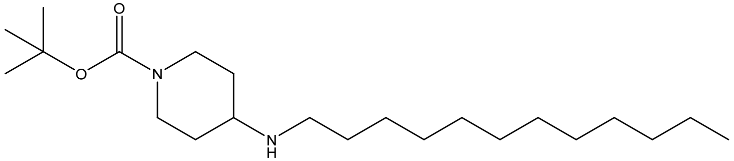	$C_{22}H_{44}N_2O_2$	368.34	3
**4c**	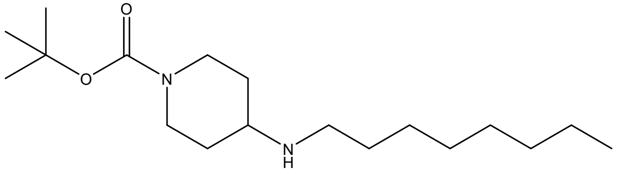	$C_{18}H_{36}N_2O_2$	312.28	75
**4d**	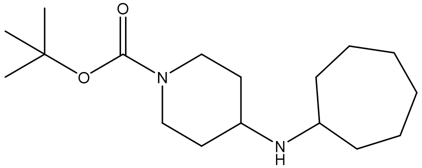	$C_{17}H_{32}N_2O_2$	296.24	>200
**4e**	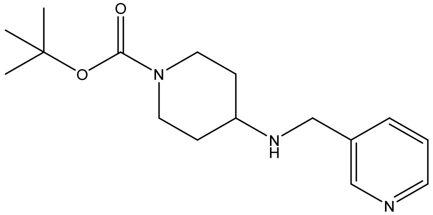	$C_{16}H_{25}N_3O_2$	291.19	>200
**4f**	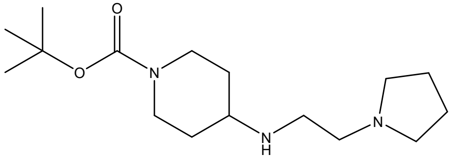	$C_{16}H_{31}N_3O_2$	297.44	>200
**5a**	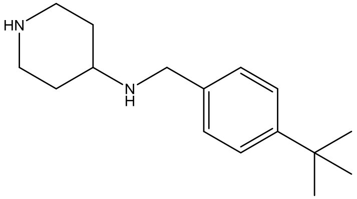	$C_{16}H_{26}N_2$	246.21	150

Name	Structure	Formula	Molecular weight [g/mol]	MIC [µg/mL]
**5b**	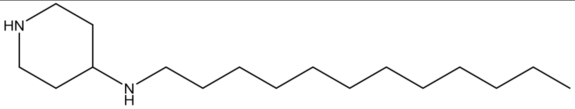	$C_{17}H_{36}N_2$	268.29	5
**5c**	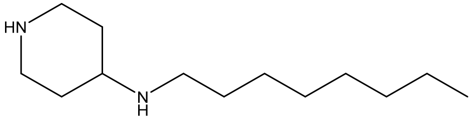	$C_{13}H_{28}N_2$	212.23	>200
**5e**	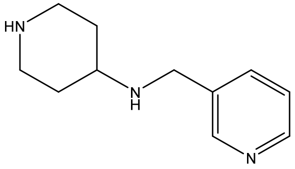	$C_{11}H_{17}N_3$	191.14	>200
**5f**	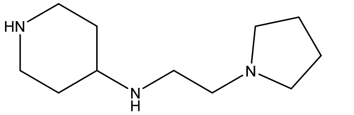	$C_{11}H_{23}N_3$	197.19	>200
**6a**	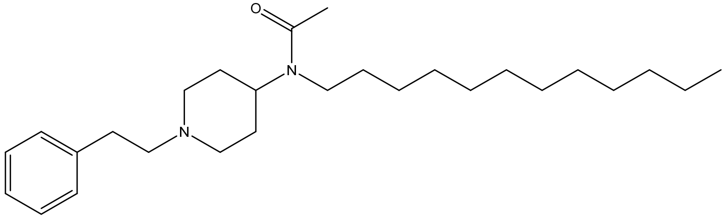	$C_{27}H_{46}N_2O$	414.36	75
**6b**	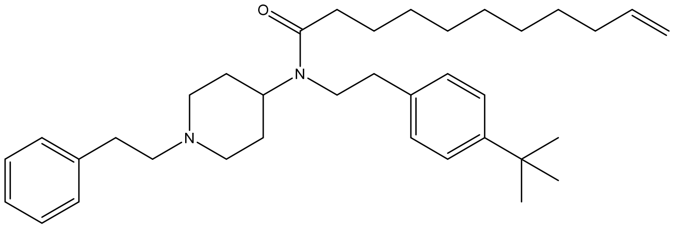	$C_{36}H_{54}N_2O$	530.42	>200

Name	Structure	Formula	Molecular weight [g/mol]	MIC [µg/mL]
**6c**	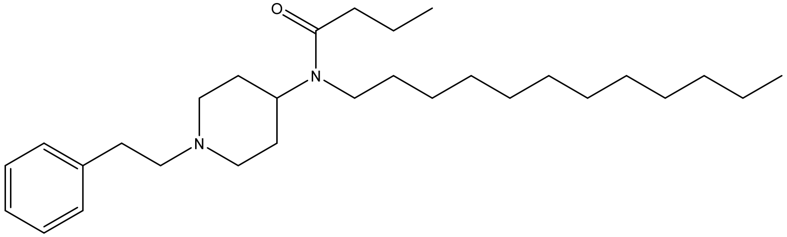	$C_{29}H_{50}N_2O$	442.39	>200
**6d**	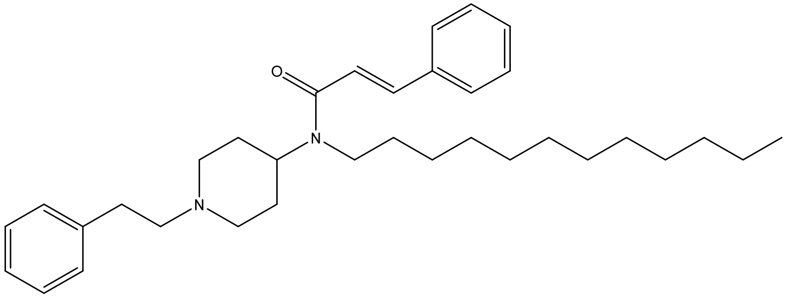	$C_{34}H_{50}N_2O$	502.39	>200
**6e**	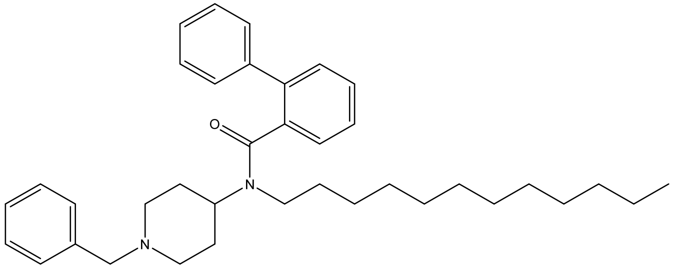	$C_{37}H_{50}N_2O$	538.39	>200
**6f**	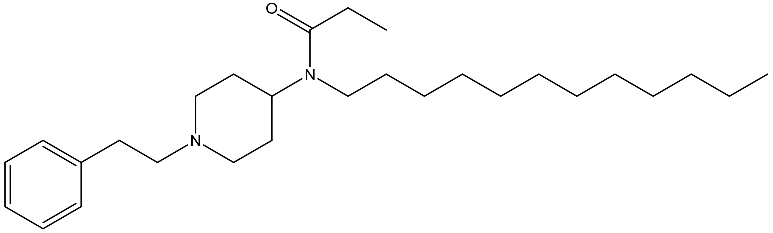	$C_{28}H_{48}N_2O$	428.38	>200

Name	Structure	Formula	Molecular weight [g/mol]	MIC [μg/mL]
**7a**	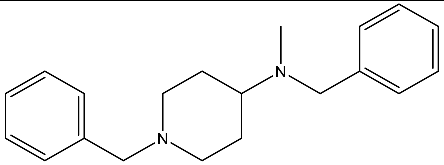	$C_{20}H_{26}N_2$	294.21	>200
**7b**	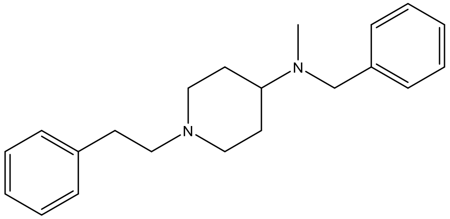	$C_{21}H_{28}N_2$	308.47	>200
**8d**	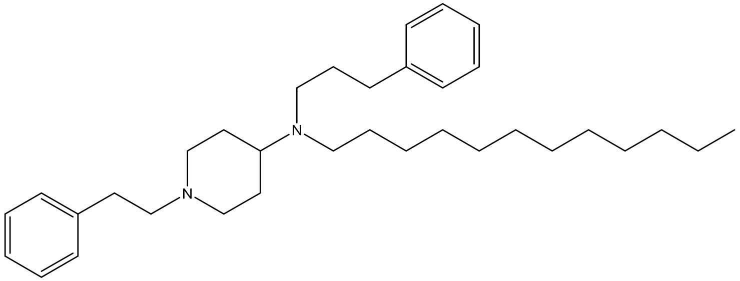	$C_{34}H_{54}N_2$	490.43	75
**8e**	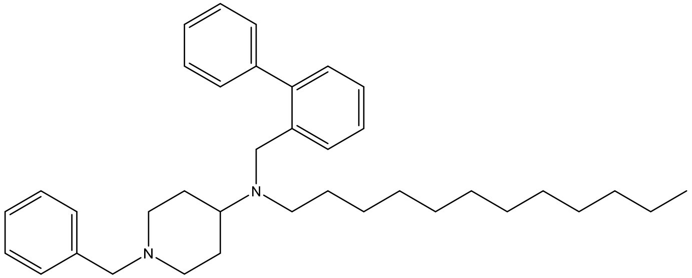	$C_{37}H_{52}N_2$	524.41	>200
