# Supplementary material for: Synthesis, Biological Evaluation, and Structure–Activity Relationships of 4-Aminopiperidines as Novel Antifungal Agents Targeting Ergosterol Biosynthesis
Source: Molecules. 2021 Nov 28;26(23):7208. doi: 10.3390/molecules26237208 (PMC8658910; doi:10.3390/molecules26237208)
Supplement: Supplementary file 1 [file molecules-26-07208-s001.zip › Supporting Information Table S2.pdf]

Supporting Information Table S2. Identification of the target enzyme(s) in cholesterol biosynthesis. Mainly accumulating sterols under incubation at different concentrations, and identified target enzymes; () trivial name; n.d. not determined

Compound	Mainly accumulating sterol(s)	Inhibited enzyme(s)
**Amorolfine hydrochloride**	**0.1 µM**: cholesta-8,14-dien-3β-ol<br>**1 µM**: cholesta-8,14-dien-3β-ol<br>**10 µM:** cholesta-8,14-dien-3β-ol	**0.1 µM:** sterol C14-reductase, sterol C8-isomerase<br>**1 µM:** sterol C14-reductase, sterol C8-isomerase<br>**10 µM:** sterol C14-reductase, sterol C8-isomerase
**2b**	**0.1 µM:** cholesta-8,14-dien-3β-ol<br>**1 µM:** cholesta-8,14-dien-3β-ol, cholesta-8-en-3β-ol (zymostenol), cholesta-5,7-dien-3β-ol (7-dehydrocholesterol), cholesta-7-en-3β-ol (lathosterol);<br>**10 µM:** toxic	**0.1 µM**: sterol C14-reductase, sterol C8-isomerase<br>**1 µM:** multi-enzyme inhibition<br>**10 µM:** n.d.
**2c**	**0.1 µM:** cholesta-8,14-dien-3β-ol, cholesta-8-en-3β-ol (zymostenol), cholesta-5,7-dien-3β-ol (7-dehydrocholesterol), cholesta-7-en-3β-ol (lathosterol)<br>**1 µM:** cholesta-8,14-dien-3β-ol, cholesta-8-en-3β-ol (zymostenol), cholesta-5,7-dien-3β-ol (7-dehydrocholesterol), cholesta-7-en-3β-ol (lathosterol);<br>**10 µM:** cholesta-8,14-dien-3β-ol, cholesta-8-en-3β-ol (zymostenol)	**0.1 µM:** multi-enzyme inhibition<br>**1 µM:** multi-enzyme inhibition<br>**10 µM:** sterol C14-reductase, sterol C8-isomerase
**2f**	**0.1 µM**: cholesta-8,14-dien-3β-ol, cholesta-8-en-3β-ol (zymostenol)<br>**21 µM:** cholesta-8,14-dien-3β-ol, cholesta-8-en-3β-ol (zymostenol), cholesta-5,7-dien-3β-ol (7-dehydrocholesterol), cholesta-7-en-3β-ol (lathosterol);<br>**10 µM:** cholesta-8,14-dien-3β-ol, cholesta-8-en-3β-ol (zymostenol)	**0.1 µM:** sterol C14-reductase, sterol C8-isomerase<br>**1 µM:** multi-enzyme inhibition<br>**10 µM:** sterol C14-reductase, sterol C8-isomerase
**2g**	**0.1 µM:** no accumulation;<br>**1 µM:** no accumulation;<br>**10 µM:** no accumulation	**0.1 µM:** n.d.<br>**1 µM:** n.d.<br>**10 µM:** n.d.
**3b**	**0.1 µM:** no accumulation;<br>**1 µM:** cholesta-8,14-dien-3β-ol, cholesta-8-en-3β-ol (zymostenol), cholesta-5,7-dien-3β-ol (7-dehydrocholesterol), cholesta-7-en-3β-ol (lathosterol);<br>**10 µM:** toxic	**0.1 µM:** n.d.<br>**1 µM:** multi-enzyme inhibition<br>**10 µM:** n.d.
**4b**	**0.1 µM:** no accumulation;<br>**1 µM:** cholesta-8,14-dien-3β-ol, cholesta-8-en-3β-ol (zymostenol), cholesta-5,7-dien-3β-ol (7-dehydrocholesterol), cholesta-7-en-3β-ol (lathosterol);<br>**10 µM:** toxic	**0.1 µM:** n.d.<br>**1 µM:** multi-enzyme inhibition<br>**10 µM:** n.d.
**4c**	**0.1 µM:** no accumulation;<br>**1 µM**: cholesta-8,14-dien-3β-ol;<br>**10 µM:** cholesta-8,14-dien-3β-ol	**0.1 µM:** n.d.<br>**1 µM:** sterol C14-reductase, sterol C8-isomerase<br>**10 µM:** sterol C14-reductase, sterol C8-isomerase
**5b**	**0.1 µM:** no accumulation<br>**1 µM:** cholesta-8,14-dien-3β-ol, cholesta-8-en-3β-ol (zymostenol), cholesta-5,7-dien-3β-ol (7-dehydrocholesterol), cholesta-7-en-3β-ol (lathosterol);<br>**10 µM:** toxic	**0.1 µM:** n.d.<br>**1 µM:** multi-enzyme inhibition<br>**10 µM:** n.d.
**6b**	**0.1 µM:** cholesta-8-en-3β-ol (zymostenol), cholesta-7-en-3β-ol (lathosterol)<br>**1 µM:** cholesta-8-en-3β-ol (zymostenol), cholesta-7-en-3β-ol (lathosterol)<br>**10 µM:** cholesta-8-en-3β-ol (zymostenol), cholesta-7-en-3β-ol (lathosterol)	**0.1 µM:** multi-enzyme inhibition<br>**1 µM:** multi-enzyme inhibition<br>**10 µM:** multi-enzyme inhibition
