# Supplementary material for: Synthesis, Biological Evaluation, and Structure–Activity Relationships of 4-Aminopiperidines as Novel Antifungal Agents Targeting Ergosterol Biosynthesis
Source: Molecules. 2021 Nov 28;26(23):7208. doi: 10.3390/molecules26237208 (PMC8658910; doi:10.3390/molecules26237208)
Supplement: Supplementary file 1 [file molecules-26-07208-s001.zip › Supporting Information Table S3.pdf]

Supporting Information Table S3: Fungal strains used in this study.

species	strain ID
Aspergillus fumigatus	A65<br>R16=ATTC46645<br>900566/2016<br>901580/2013<br>920895 (I)/2015<br>R7=ATCC204305
Aspergillus terreus	T155<br>T65<br>T73<br>T36<br>T98<br>R15= MYA3633
Aspergillus flavus	508978/2014<br>902400/2012=FL50<br>901074/2013<br>901104/2013<br>900151/2012=FL29<br>R8= ATCC 204304
Mucormycetes	
Rhizopus microsporus	AS73
Rhizopus arrhizus	LunD3
Rhizopus microsporus	11 14
Lichtheimia ramosa	11 11
Apophysomyces elegans	VPCI 14
Mucor circinelloides	VPCI 19
Syncephalastrum racemosum	VPCI 8
Lichtheimia corymbifera	07 10
Candida albicans	17046452<br>17046508<br>17047778<br>19082249<br>19082941<br>CR2=ATCC 90028
Candida glabrata	17043820<br>17132978<br>18094734<br>19101275<br>19161510<br>CG2<br>CR4=ATTC 90030
Candida tropicalis	17090801<br>19082746<br>19161237/2016<br>24453/2016<br>27612<br>CR8 = ATCC 90874
Candida krusei	80<br>623/5<br>BK60<br>14<br>461298/2016<br>CR9=ATCC 6258
Candida auris	Sc1
Yarrowia lipolytica	78-003
